# Phenotyping, genetics, and “-omics” approaches to unravel and introgress enhanced resistance against apple scab (*Venturia inaequalis*) in apple cultivars (*Malus* × *domestica*)

**DOI:** 10.1093/hr/uhae002

**Published:** 2024-01-10

**Authors:** Anže Švara, Nico De Storme, Sebastien Carpentier, Wannes Keulemans, Barbara De Coninck

**Affiliations:** Laboratory for Plant Genetics and Crop Improvement, Division of Crop Biotechnics, KU Leuven Plant Institute, Willem de Croylaan 42, 3001 Leuven, Belgium; KU Leuven Plant Institute, KU Leuven 3001 Leuven, Belgium; Laboratory for Plant Genetics and Crop Improvement, Division of Crop Biotechnics, KU Leuven Plant Institute, Willem de Croylaan 42, 3001 Leuven, Belgium; KU Leuven Plant Institute, KU Leuven 3001 Leuven, Belgium; Laboratory of Tropical Crop Improvement, Division of Crop Biotechnics, KU Leuven, Willem de Croylaan 42, 3001 Leuven, Belgium; Genetic resources, Bioversity International, Willem de Croylaan 42, 3001 Leuven, Belgium; KU Leuven Plant Institute, KU Leuven 3001 Leuven, Belgium; Laboratory for Plant Genetics and Crop Improvement, Division of Crop Biotechnics, KU Leuven Plant Institute, Willem de Croylaan 42, 3001 Leuven, Belgium; KU Leuven Plant Institute, KU Leuven 3001 Leuven, Belgium; Laboratory of Plant Health and Protection, Division of Crop Biotechnics, KU Leuven, Willem de Croylaan 42, 3001 Leuven, Belgium; Laboratory for Plant Genetics and Crop Improvement, Division of Crop Biotechnics, KU Leuven Plant Institute, Willem de Croylaan 42, 3001 Leuven, Belgium

## Abstract

Apple scab disease, caused by the fungus *Venturia inaequalis*, endangers commercial apple production globally. It is predominantly managed by frequent fungicide sprays that can harm the environment and promote the development of fungicide-resistant strains. Cultivation of scab-resistant cultivars harboring diverse qualitative *Rvi* resistance loci and quantitative trait loci associated with scab resistance could reduce the chemical footprint. A comprehensive understanding of the host–pathogen interaction is, however, needed to efficiently breed cultivars with enhanced resistance against a variety of pathogenic strains. Breeding efforts should not only encompass pyramiding of *Rvi* loci and their corresponding resistance alleles that directly or indirectly recognize pathogen effectors, but should also integrate genes that contribute to effective downstream defense mechanisms. This review provides an overview of the phenotypic and genetic aspects of apple scab resistance, and currently known corresponding defense mechanisms. Implementation of recent “-omics” approaches has provided insights into the complex network of physiological, molecular, and signaling processes that occur before and upon scab infection, thereby revealing the importance of both constitutive and induced defense mechanisms. Based on the current knowledge, we outline advances toward more efficient introgression of enhanced scab resistance into novel apple cultivars by conventional breeding or genetic modification techniques. However, additional studies integrating different “-omics” approaches combined with functional studies will be necessary to unravel effective defense mechanisms as well as key regulatory genes underpinning scab resistance in apple. This crucial information will set the stage for successful knowledge-based breeding for enhanced scab resistance.

## Introduction to apple scab resistance

Domesticated apple (*Malus* × *domestica* Borkh.) has the highest economic value among fruit crops grown in the temperate climate zone, although it is endangered by both biotic and abiotic factors [1]. Among these factors, the hemi-biotrophic ascomycete fungus *Venturia inaequalis* causing apple scab is considered the economically most important pathogen of apple [2], as it is associated with costly and frequent fungicide applications [3]. *Venturia inaequalis* primarily affects leaves and fruit by forming velvety sporulating, chlorotic, and necrotic lesions that can in turn result in reduced flower bud formation affecting both yield and produce quality [4]. Approximately 75% of the crop protection products used in apple cultivation is dedicated to the management of fungal diseases, with more than half of this use specifically targeted at combating apple scab. As a result, scab management can cost over a thousand dollars per hectare [5, 6] and inadequately managed scab infections can lead to economic losses amounting up to 70% or more [7, 8]. The highly damaging nature of this fungal disease is fostered by conducive climatic conditions, i.e. particularly humidity, together with the development of virulent pathogenic strains and high prevalence of scab-susceptible commercial cultivars [2, 9, 10].

Prevention of apple scab-related losses is largely based on fungicide treatments with yearly averages of ~15 applications per growing season [2, 9, 11]. Despite continuous efforts to optimize treatments via improved warning systems and phytosanitary leaf litter management, selection pressure has resulted in the development of fungicide-resistant *V. inaequalis* strains. For example, several fungicides with active substances such as benomyl or quinone outside inhibitors were shown to be ineffective within a decade of their use [12]. Moreover, chemical control of apple scab is subject to increasingly stricter regulations, driven by the growing awareness of pesticide-related health and environmental risks, leading to a strong reduction in the number of permitted and newly approved active substances [13]. As a result, complementary approaches need to be explored and devised to mitigate scab infections in apple cultivation.

The development and implementation of premium quality cultivars with enhanced resistance against *V. inaequalis* could assist in more sustainable scab management [9, 10]. Currently, conventional breeding approaches for scab resistance mainly involve the integration of qualitative resistance loci, i.e. harboring major-effect *R* alleles, from different *Malus* species into new commercial cultivars [14–17]. When such loci result in qualitative resistance, also referred to as gene-for-gene (GfG) or vertical resistance, against the majority of *V. inaequalis* races, they are in literature referred to as *Rvi* genes with an additional number referring to the compatible interaction with a specific *V. inaequalis* race lacking the corresponding *Avr* (avirulence) gene [9]. However, as actual causative genes and alleles for the majority of *Rvi* loci, except for *Rvi6* and *Rvi4*, have not been identified yet, we will term them “*Rvi* loci”. So far, 18 putatively unique *Rvi* loci harboring qualitative resistance alleles (Suppl. Table 1) have been identified in different wild *Malus* species and cultivars, and these map onto 11 of the 17 chromosomes of the apple genome [18–22]. Particularly, the *Rvi6* locus harboring the *HcrVf2* resistance allele, originating from the wild species accession *Malus floribunda* 821, has been crossed into over 90% of scab-resistant cultivars [10, 23], such as ‘Bonita’, ‘Florina’, ‘Fujion’, and ‘Topaz’. However, scab-resistant cultivars are currently cultivated to a lesser extent compared to susceptible cultivars, and are often limited to organic production, as many of these cultivars exhibit inferior tree architecture, fruit morphological quality, and organoleptic properties, rendering them less suitable for commercial apple production [24].

Generally, *R* genes encode either intracellular or sometimes transmembrane receptor proteins, which directly or indirectly recognize pathogen effectors, termed Avr proteins [25, 26]. This recognition triggers a signaling cascade, which activates effector triggered immunity (ETI) [26, 27], preventing pathogen proliferation. In many cases, ETI entails a local hypersensitive response (HR) resulting in cell death, and hence it is effective against biotrophic pathogens [26, 28–31]. Subsequent downstream defense signaling can trigger accumulation of specialized metabolites, pathogenesis-related (PR) proteins, or reactive oxygen species detoxifying enzymes, further contributing to the defense response [32]. However, the range of pathogen effectors recognized by the major-effect R proteins can vary greatly, making pathogenic strain-specificity an inherent feature of ETI [30]. Moreover, under specific environmental conditions and in specific host genotypes, *Rvi* loci may result in a continuous output ranging from complete susceptibility to resistance, i.e. as reflected by heavy sporulation to no sporulation, respectively [33, 34]. Also, R proteins exert high selection pressure on the pathogen and therefore are often non-durable. As observed for several apple cultivars, scab resistance mediated by an *Rvi* locus putatively harboring a single resistance allele can be overcome by the pathogen even within the timeframe of one single decade [9, 35, 36]. Integration of multiple effective *Rvi* loci in a single genotype has been considered to improve the durability of resistance. However, knowledge on the most efficient and compatible *Rvi* combinations and insights into the mechanistic basis of each allele of the *Rvi* loci could streamline this approach.

Some apple cultivars, such as ‘Common Antonovka’, ‘Président Roulin’, ‘Discovery’, TN10-8, ‘Durello di Forli’, and ‘Dülmener Rosenapfel’, exhibit quantitative resistance (section 3.2.) [10, 28, 30, 37–47]. This type of resistance is typically controlled by multiple alleles involved in a range of different/complementary mechanisms each contributing to a certain extent to the cumulative build-up of scab resistance and thus individually only exert a low selection pressure on the pathogen [10, 30, 37–47] (Suppl. Table 2). It should be distinguished from partial resistance based on a single defeated major-effect *R* allele with a residual effect [9, 48]. Quantitative resistance typically entails more moderate- and minor-effect alleles within quantitative resistance loci (QRLs), including those that upon infection trigger broad-spectrum pattern triggered immunity (PTI) [28]. In particular, PTI is triggered by pathogen- or microbe-associated molecular patterns (PAMPs and MAMPs, respectively), initiating a downstream signaling cascade via mitogen-activated protein kinase cascade, leading to changes in plant defense hormones, production of reactive oxygen species, accumulation of PR proteins and specialized metabolites, and reinforcement of the plant cell wall [30, 48–51]. Genes involved in such quantitative resistance mechanisms typically have multiple alleles that may contribute differently to the strength of the response [48]. As a consequence, quantitative scab resistance exhibits a continuous phenotypic distribution ranging from susceptible to resistant in segregating populations [49]. Moreover, the strength of quantitative resistance is also influenced by environmental factors with variations in temperature and rainfall during spring and early summer strongly interfering with overall resistance [47, 52], but is generally considered as (equally) effective against all strains of a pathogen and is thus often referred to as horizontal resistance [53]. Although the resistance of apple to *V. inaequalis* can be described by the two aforementioned resistance categories, in reality, the two resistance types and their respective underlying mechanisms sometimes resemble each other [28].

**Figure 1:**
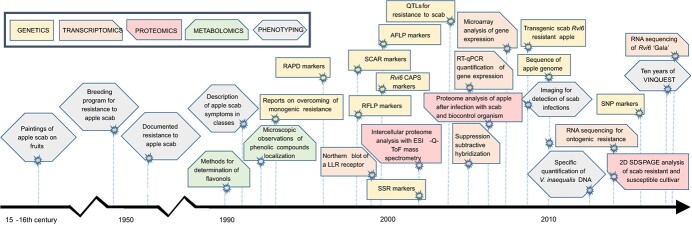
Historical overview of approaches to study the *Malus* × *domestica*–*Venturia inaequalis* interaction, in order to elucidate mechanisms underlying resistance mechanisms to apple scab. VINQUEST refers to the project collecting information on the geographical distribution of *V. inaequalis* populations with specific (a)virulence genes [43, 54]

This review assesses the current stage of knowledge on phenotypic variation in resistance of apple plants against infection with different *V. inaequalis* strains harboring different qualitative *Rvi* scab resistance loci or QRLs, the underlying genetic factors, and their molecular responses studied through “-omics”. This is enabled by a variety of approaches that have been implemented to unravel different aspects of the *M.* × *domestica*–*V. inaequalis* interaction (Fig. 1). Furthermore, we discuss the implications of the enhancement of scab resistance by introduction of different resistance loci and the contributing resistance alleles into commercial apple cultivars via conventional cross-breeding or via genetic modification techniques (e.g. genome editing and cisgenesis). Finally, we discuss the existing knowledge gaps that impair practical introduction of robust and enhanced scab resistance in apple. Enhancing of scab resistance necessitates an in-depth comprehensive understanding of the *M.* × *domestica*–*V. inaequalis* host–pathogen interaction and host defense responses, as well as of how environmental conditions impact these mechanisms. This not only entails identification of major-effect *R* alleles and their interactions with different *V. inaequalis* races, which has already been reviewed earlier [9, 24, 43], but also involves gaining novel insights into minor-effect genes underlying quantitative resistance.

## Phenotypic evaluation of apple response to scab infections

**Figure 2:**
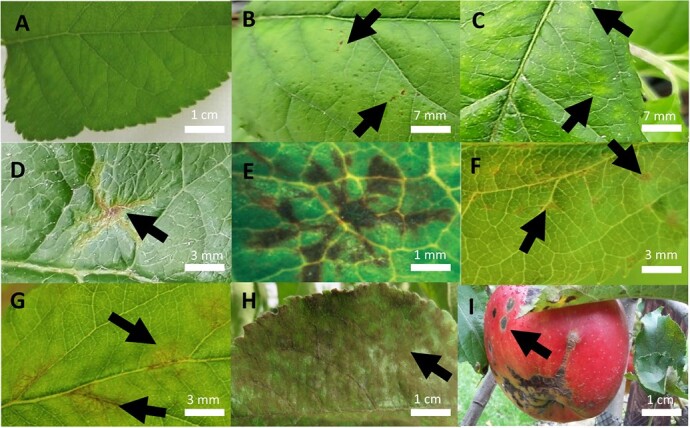
Overview of apple scab (*Venturia inaequalis*) symptoms on the adaxial surface of the apple leaf and characteristic symptoms on apple fruit: (**A**) Chevalier class 0 with no symptoms; (**B**) class 1 with pinpoint pits; (**C**) class 2 showing chlorosis, (**D**) radial-shaped necrosis on a leaf of a scab-resistant cultivar, and (**E**) stellate necrosis observed on an apple genotype harboring the *Rvi2* locus (with permission [9, 58]); (**F**) class 3a with necrosis, chlorosis, and occasionally light sporulation; (**G**) class 3b showing chlorosis, necrosis, and more distinct sporulation; (**H**) class 4 with severe sporulation; (**I**) scab symptoms on fruit. Pictures **A**–**D**, **F**, and **G** were taken in a greenhouse inoculation experiment using ‘Makali’ and ‘Gala’ plants inoculated with *V. inaequalis* race (1) isolate 104, and pictures **H** and **I** were taken in a ‘Jonagold’ orchard.

In 1902, Rudolf Aderhold artificially inoculated 160 apple accessions with *V. inaequalis* spores and observed that some of them express more severe symptoms and thus higher levels of scab susceptibility than others [55, 56]. In general, the most obvious apple scab symptoms consist of black, gray, or brown lesions developing mainly on leaves, fruit, and sometimes on other green aerial organs, including petioles, shoots, and bud scales [57]. Depending on the level of scab resistance, apple cultivars can show a variable expression of symptoms on these organs (Fig. 2) [10]. However, selecting less susceptible cultivars with low levels or absence of sporulation or specific defense reactions in forms of chlorosis and necrosis, requires a robust and accurate system for disease symptom evaluation. This evaluation system, based on artificial *V. inaequalis* inoculation under controlled conditions (for details see section 2.1.3.), should enable discrimination among the subtle reactions indicating whether pathogen infection, colonization, or reproduction is interrupted.

### Disease symptoms and their evaluation

#### Symptoms on leaves

Apple cultivars that are susceptible to *V. inaequalis*, such as ‘Gala’, typically develop sporulating, chlorotic, and necrotic lesions within the first 10 days post-inoculation (dpi) with *V. inaequalis*. These symptoms refer to the successful formation of asexual reproductive structures, i.e. conidiophores carrying conidia. Over time, the complete leaf is covered with sporulating lesions (Fig. 2G and H), subsequently resulting in leaf curling and drying, and even premature leaf drop [57, 59, 60]. The latter three symptom types are indirect as they result from increased sporulation causing down-regulation of genes encoding for proteins involved in photosynthesis followed by an uncontrolled collapse and death of cells [2, 60, 61].

Apple varieties that harbor vertical resistance typically exhibit symptoms that are often linked to a specific resistance allele of the *Rvi* locus (Suppl. Table 1). The first macroscopic symptoms that appear in some monogenic resistant cultivars within 3 dpi [62], as for instance in those carrying *Rvi5*, are characterized by pinpoint pits on the leaf surface [63] (Fig. 2B). These dark depressions in the leaf surface have a diameter of <1 mm and likely result from a hypersensitive response (HR) [59] leading to the collapse of cells in and around the penetration site [64]. The HR can be accompanied by the degradation of chlorophyll leading to chlorotic spots surrounding the collapsed cells (Fig. 2C) [60]. Other *Rvi* loci, such as *Rvi2*, result in a limited subcuticular growth of stromae and hyphae that leads to the formation of irregular star-shaped necrotic lesions (Fig. 2D), often referred to as “stellate necrosis” (Fig. 2E) [9, 58]**,** suggesting a delayed defense response. In the case of *Rvi2,* this type of necrosis appears ~4–6 days after inoculation [64–67]. Further delays in the defense response can result in the appearance of small necrotic zones occurring at 7 dpi, as for example in the case of *Rvi15* [68] (Fig. 2F). A combination of these symptoms can appear in a plant carrying resistance loci, such as *Rvi6* [69], and this can be additionally influenced by ontogenic resistance attributed to increasing age of apple organs (section 2.2) [2, 70].

In most cultivars, the described apple scab symptoms develop on the adaxial side of immature leaves, but occasionally develop also on the abaxial side. In greenhouse experiments, the appearance of abaxial symptoms is generally delayed compared to adaxial ones [71]. This may be caused by the altered morphology of the abaxial leaf side with a high trichome density and a variable cuticular wax composition and thickness resulting in a lower wettability and a prolonged physical protection [72, 73]. Although apple scab symptoms can be expressed both on the ad- and abaxial leaf side, adaxial surface inoculation and evaluation is the preferred approach for determining disease symptoms.

#### Symptoms on fruit

Apple scab lesions on fruit can appear everywhere on the young fruit surface, and in particular at the calyx or lateral exocarp fruit sides. When older, fruit lesions turn dark brown, become corky, and impede further fruit growth in the affected area (Fig. 2I). As such, the lesions can lead to malformed fruit and altered fruit size or shape. Eventually, affected fruit can develop cracks, making them more susceptible to secondary infections [57, 74]. Similar to leaves, fruit exhibit ontogenic resistance with growing fruitlets being more susceptible than older fruit [75]. Strikingly, the development of fruit symptoms sometimes only weakly correlates with leaf symptoms, suggesting that the defense response is highly tissue specific. This is further supported by the fact that cultivars exhibiting the most severe leaf symptoms are different from cultivars with the most severe fruit symptoms [37, 76].

#### Assessment of symptom severity variation on leaves

The first step toward the use of apple genotypes in apple scab resistance breeding is the accurate determination of their resistance/susceptibility. Until now, the evaluation was based on the classification of the macroscopic leaf symptoms into specific classes, and for this several systems are available [10, 14, 59, 77]. Among these systems, the classification of the disease symptoms into six Chevalier classes (0, 1, 2, 3a, 3b, and 4; Fig. 2) based on the reactions observed in a progeny from a cross between a susceptible commercial cultivar and an *Rvi6*-harboring cultivar [59], inoculated with *V. inaequalis* race (1), is still widely used today [78, 79]. Isolates of *V. inaequalis* race (1) have a widespread distribution, as the majority, i.e. 18 out of 23, of standard *V. inaequalis* isolates were designated as race (1) and are virulent to ‘Golden Delicious’ [69], which harbors the *Rvi1* locus. Race 1 isolates can overcome *Rvi1* and have been extensively studied, which makes this an important reference race to advance our understanding of the basis of scab resistance [9]. In interaction with a single *Rvi* locus, as for example with *Rvi6*, race 1 isolates can result in a plethora of defense reactions [69]. Mostly, they show classes ranging from 0 to 3a describing leaves without or with only slight sporulation and thus are considered resistant [59]. However, also leaves and plants with distinct sporulation covering less or more than 50% of the leaf surface that belong to classes 3b or 4, respectively, can be observed and are considered weakly and completely susceptible, respectively [59].

Accurate evaluation of scab symptoms requires phenotyping based on a continuous scale as even among genotypes harboring the same *Rvi* locus can vary significantly and be expressed as a continuum ranging from low, i.e. absence of symptoms, to high, i.e. significant sporulation, depending on the specific *V. inaequalis* strain, genetic background of the plant host, or the environment. For instance, plants carrying *Rvi3* and *Rvi6* are classified in the resistant classes ranging from 0 to 3a toward the majority of *V. inaequalis* strains, but can be classified into the susceptible classes 3b or 4, depending on plant’s genetic background (e.g. presence of genes that weaken the defense reaction), infection conditions, and the aggressiveness of *V. inaequalis* strains [9, 33, 34, 59]. Accurate assessment of symptom severity is further complicated by environmental conditions such as leaf wetness, i.e. with an optimal duration of leaf wetness of ~20 h, and temperature, i.e. with an optimum between 16 and 20°C [80]. As a consequence, symptoms observed under greenhouse conditions can differ from those in the orchard, as was for example observed for plants carrying *Rvi6* [10, 81]. Therefore, greenhouse observations should consistently be validated under field conditions. The continuous distribution and assessment of the severity of disease symptoms across individuals with quantitative resistance in a population can be performed by using for example microscopy or molecular techniques [4, 82–89]. Particularly during early infection stages, including the pre-penetration stage, appressoria formation, subcuticular stroma formation, and the growth of subcuticular runner hyphae, accurate quantitative and qualitative evaluation of the susceptibility degree of apple to scab can be performed via microscopic analysis of fungal growth. Detection and quantification of fungal structures is thereby mainly achieved using aniline blue or trypan blue staining of fungal cell walls and callose [90], or by scanning and transmission electron microscopy [90–93]. Alternatively, the growth of the fungus can be validated by molecular quantification of fungal DNA in or on the leaf using a *V. inaequalis*-specific qPCR or via loop-mediated isothermal amplification (LAMP) [4, 85–89].

### Ontogenic resistance

Ontogenic scab resistance is characterized by development-dependent resistance whereby mature organs are typically resistant or less susceptible than younger ones [94]. While ontogenic resistance has been observed in both apple leaves and fruit, with a considerable variation among different genotypes [2, 71, 94, 95], research on the mechanisms of ontogenic resistance only focus on leaves [2, 10, 95]. Ontogenic resistance is considered stable and durable over years and acts against all *V. inaequalis* strains [58]. However, it provides only partial protection as the fungus can successfully penetrate the cuticle and infect leaves or fruit at a younger stage, remain latent and quiescent for a specific period of time, and then again resume mycelial growth in older leaves to complete its life cycle at the onset of senescence [10, 70, 96]. Nevertheless, in leaves with increasing age, incidence and area of scab lesions are reduced in comparison to younger leaves. MacHardy [2] stated three mechanisms that putatively underlie ontogenic resistance and act against the pathogen upon cuticle penetration in older leaves. These mechanisms include (1) decreased cellular tissue pH, (2) inactivation of cell wall degrading enzymes, and (3) production of metabolites with antimicrobial activity [2, 93, 94]. Crushed young leaves have a pH around 6, which suppresses or inhibits the activity of defense proteins. When leaves mature, the pH decreases to around 5, which enables increased activity of for example polyphenol oxidase, an enzyme that converts phenolics to *o*-quinones degrading fungal melanoproteins, and polygalacturonase-inhibiting proteins inhibiting plant cell wall degradation [2]. Finally, as the aging in mature and senescent leaves progress, the pH returns to 6, corresponding with a recontinuation of fungal growth with subsequent appearance of late disease symptoms [2, 94, 97]. Also, studies on older leaves of ‘Golden Delicious’ showed increased expression of putative resistance-related genes [94] (section 4.1). All these insights illustrate the mechanistic and molecular basis underlying ontogenic scab resistance.

## Genetics of scab resistance

### Qualitative resistance by major-effect resistance alleles

Qualitative scab resistance is conferred by loci harboring major-effect *R* alleles that can cover a continuum from complete resistance to partial resistance [9, 28, 29, 48], with the strength of their effects conditioned by the environment and pathogenic strains present [9, 28, 48, 98]. In this review, we restrict the term *R* (and *Rvi*) genes and their corresponding resistance alleles exclusively to the major-effect genes and alleles involved in well-documented cases of ETI (Suppl. Table 1). Eighteen such *R* alleles associated with *V. inaequalis* resistance are present in various germplasm accessions [9, 10], and all of the characterized resistance alleles of the *Rvi* genes are dominant [9], although for most of these genes the function of the associated alleles is yet unknown [18–21].

Many other genes formerly recognized as major-effect *R* genes are excluded from the *Rvi* nomenclature (www.vinquest.ch) [9, 43, 54]. In the past, these loci, such as *Vt57*, *Vs/Vsv*, and *Vd3*, e.g. found in ‘Boskoop’, ‘Bramley’, ‘Cox’s Orange Pippin’, ‘Spartan’, and ‘Worcester’, may have offered resistance against a wide spectrum of *V. inaequalis* strains, which has been putatively broken by many of these strains, although no solid evidence is available. However, since they have been widely overcome by virulent strains [9, 10, 43, 69, 99–102] and are only effective against a small number of the existing races, these genes are of lower importance for resistance breeding [9, 43, 64, 67, 98, 103–111].

Similarly to *Rvi* genes, ~600 *V. inaequalis* “candidate effector genes” have been identified [74, 98, 112–115]. However, these effector genes and proteins have not been functionally characterized [116, 117]. This information together with knowledge on their specific recognition by potential R proteins and pathways, however, would be highly relevant for gaining more insight into the molecular basis of the effector–receptor interactions and their functional role in apple scab resistance [29, 118, 119].

#### Genetic structure of *Rvi* loci reveals presence of multiple genes and paralogs

**Figure 3:**
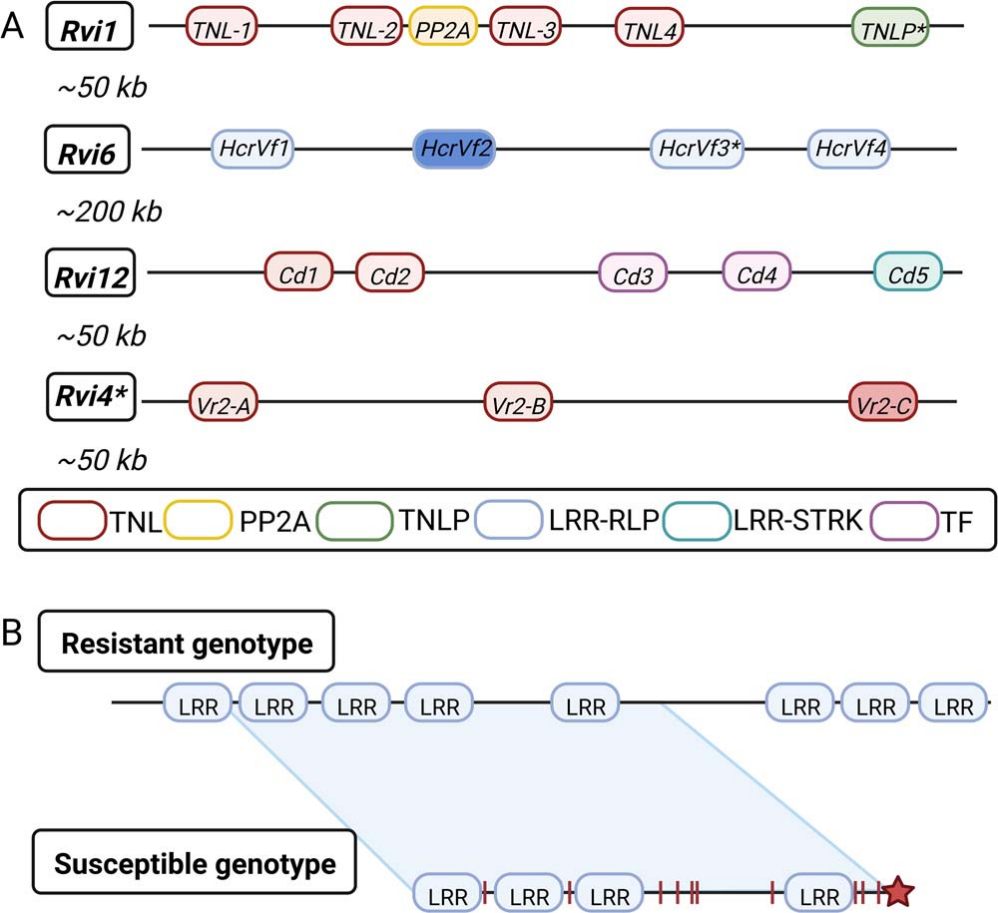
Schematic illustration of the genetic structure of the known monogenic scab resistance loci. (**A**) Genetic structure of *Rvi1*, -*6*, -*12*, and -*15* reconstructed based on previous studies [68, 121–126]. Genetic elements without a potential function in defense are not included in the scheme. Dark *HcrVf2* and *Vr2-C* alleles correlate with the absence of sporulation. Genes with an asterisk (*) are considered non-functional, and the asterisk next to the *Rvi4* indicates that this locus was previously known as/is identical to *Rvi15* [127]. (**B**) Illustration of the genetic structure of an *Rvi* locus from a scab-resistant and -susceptible genotype, showing lack of specific *LRR* paralogs in the susceptible allelic variant, several point mutations (red vertical lines), and a putative stop codon (a dark-red star) [128]. TNL: toll/interleukin-1 receptors containing a nucleotide-binding site and leucine-rich repeat motif, PP2A: serine/threonine phosphatase 2A, TNLP: TNL pseudogene, LRR: leucine-rich repeat motif (in **B**), LRR-RLP: LRR receptor-like protein LRR-STRK: LRR receptor-like serine/threonine-protein kinase, TF: transcription factor. *Cd*, *Vr*, and *HcrVf*: names of specific groups of genes.

*Rvi* loci harbor various closely linked genes that are unknown for their role in resistance [9, 68, 120, 121]. Identification of candidate resistance alleles of *Rvi1*, *Rvi6*, *Rvi12*, and *Rvi4* loci revealed the presence of multiple paralogs (Fig. 3A). For example, the *Rvi1* locus harbors four toll/interleukin-1 receptors (TIR) containing a nucleotide-binding site (NBS) and a leucine-rich repeat (LRR) together with a TIR-NBS-LRR (TNL) pseudogene and one serine/threonine protein phosphatase 2A gene [122]. In the *Rvi6* locus, four LRR receptor-like proteins (LRR-RLP, referred to as HcrVf1–4) were detected [121], whereas in the *Rvi4* locus three TNLs were found [68, 123]. Similarly, the *Rvi12* locus harbors a gene that encodes an LRR receptor-like serine/threonine-protein kinase family protein (LRR-STRK), together with five other resistance gene analogues (RGAs), of which four are putatively disfunctional [124]. The presence of multiple paralogous copies of receptor proteins in several distinct *Rvi* loci may indicate a specific functional role of allele/protein dosage, although the exact molecular function of all these *Rvi* candidate alleles still needs to be unraveled before the relevance of multiple alleles and paralogous copies can be investigated. In addition, it is not yet clear whether other *Rvi* loci harbor gene paralogs as well, and if all genes within the *Rvi* loci are genetically linked and inherited as strict linkage blocks.

Functional and comparative sequence analysis of individual paralogs within the *Rvi6* and *Rvi15* loci revealed that the paralogs differ in their genetic structure, sequence, and in their contribution to the conferred resistance (Fig. 3B) [123, 129, 130]. Firstly, paralogs *HcrVf1*, *-2*, *-3*, and *-4* spanning the *Rvi6* locus on linkage group (LG) 1 [121, 131] differ among each other due to unique polymorphic nucleotides, a number of short random duplications or deletions, and various deletions of complete LRR copy units [132]. ‘Galaxy’ and ‘McIntosh’ plants transformed with either *HcrVf1* or *HcrVf2* showed reduced symptom development compared with wild-type plants when inoculated with a mixture of *V. inaequalis Rvi6*-avirulent isolates from races 1 to 5 (races known to be at the time avirulent to *Rvi6*), while higher susceptibility was observed in plants transformed with *HcrVf4* [130] (Suppl. Table 3). In contrast, Joshi et al. [129] observed decreased susceptibility against *Rvi6*-avirulent races only for *HcrVf2*, whereas *HcrVf1* did not confer resistance against any of the races, suggesting that in the tested genotypes *HcrVf2* is the only paralog that actually determines resistance [129]. Due to a transposon-like insertion, the *HcrVf3* paralog from *M. floribunda* 821 is considered non-functional, although no experimental evidence is available [23, 125, 130, 132, 133], and additional cultivars should be sequenced to unravel if this specific insertion occurs in all *Rvi6* genotypes. Secondly, functional characterization of the three paralogs of the *Rvi15* locus, named *Vr2-A*, *Vr2-B*, and *Vr2-C*, demonstrated that only *Vr2-C* correlates with absence of sporulation after inoculation with an *Rvi15*-avirulent mixed *V. inaequalis* field population [68, 123]. Further cloning and functional characterization of candidate alleles/paralogs and their corresponding resistance alleles in the majority of known *Rvi* loci has yet to be carried out to determine their specific role and molecular mechanistic basis in conferring scab resistance. This will be key in exploiting them for developing plants with enhanced resistance [54, 120] and could offer insights into the evolution of different paralogs in host–pathogen arms race [134].

#### Resistance gene analogues

In addition to the *Rvi* loci containing multiple genes and gene paralogs, numerous other candidate *RGAs* have been identified across the ‘Golden Delicious’ reference genome using blast and Hidden Markov Model search program for NBS domains [134–137]. Such *RGAs* can be characterized by various motifs [138], although in apple, they most commonly contain an NBS domain (868 and 1015 *RGAs*, respectively) that is most frequently associated with an LRR domain (43.4% RGAs) [134, 138]. Although they are spread over the entire genome, they are often located in the physical proximity of *Rvi* loci and are therefore often co-inherited with these *Rvi* loci [135–137]. For instance, in the resistant cultivar ‘Geneva’, five NBS-LRR *RGAs* are in a close physical proximity to the *Rvi3* resistance locus spanning a 5-cM region (2.2 Mbp) on LG4 [9, 120]. However, it is unclear whether these *RGAs* actually contribute to resistance and affect the degree of *Rvi3* resistance. Hence, additional functional elucidation of the role of *RGA*s in resistance against *V. inaequalis* and their co-localization with *Rvi* loci is required.

### Quantitative resistance loci

Apple cultivars maintained in germplasm repositories are a rich source of diverse functional alleles that are potentially involved in quantitative resistance effective against a broad range of *V. inaequalis* strains. Such resistance, which is typically controlled by a cumulative effect of a large number of genes that are located on QRLs [30, 47, 139, 140], has been observed in cultivars such as ‘Discovery’, TN10–8, and ‘President Roulin’ [10, 24, 140–142]. In addition, a large untapped potential of QRLs may exist in apple germplasm, as for 177 wild *Malus* accessions and domesticated cultivars ~37% of them had no common parents with the previously studied genotypes [143].

#### Identification of quantitative resistance loci for apple scab

**Figure 4:**
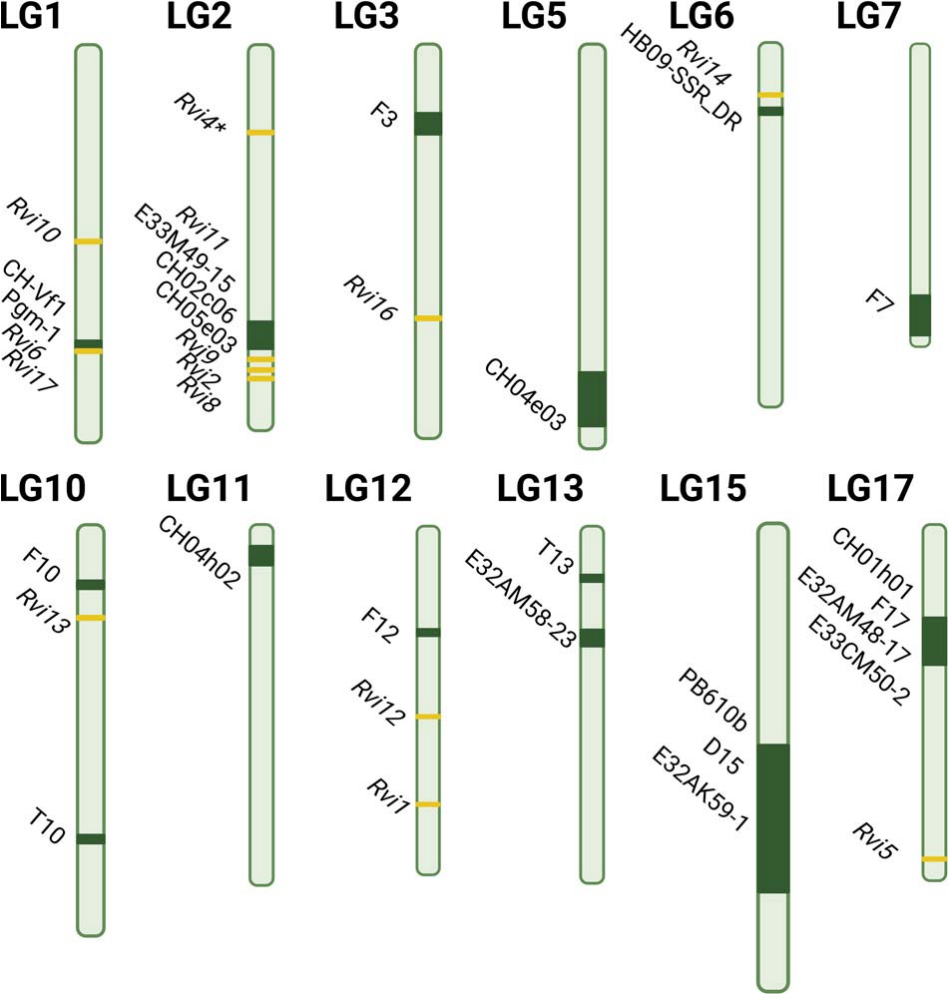
Schematic representation of the genomic position of the reported markers common to different quantitative scab resistance loci (QRL) and *Rvi* loci (dark green and yellow, respectively) on apple linkage groups. The scheme is based on the integrated consensus map from Genome Database for Rosaceae (https://www.rosaceae.org/) and on the existing data from QRL studies in apple [37, 38, 47, 140, 144]. An asterisk (*) next to *Rvi4* indicates that this locus has recently been identified as identical to *Rvi15* [127].

A handful of linkage-based mapping studies have been performed on various biparental apple seedling populations that display variability in susceptibility against different *V. inaequalis* strains to identify underlying QRLs [37, 38, 47, 140, 144]. Altogether, these mapping studies yielded 52 QRLs located on 12 LGs across the genomes of only six cultivars, ranging significantly in their contribution to the total genetic variation in *V. inaequalis* resistance, i.e. from 3.5 to 82.7% (Suppl. Table 2). Strikingly, specific QRLs are found to have a major effect on the phenotypic variation in resistance (Fig. 4), suggesting that putative *Rvi* homologs can underlie such loci [39]. However, whether or not and to what extent these alleles of the *R* genes contribute to cumulative effects of QRLs needs to be further clarified.

From a functional perspective, it is yet unclear which genes actually underlie the resistance conferred by scab QRLs. Basically, quantitative resistance may be caused by resistance alleles of the genes involved in the recognition of *V. inaequalis*, such as *Rvi* paralogs, *RGAs*, or other recognition receptors, or by signaling and downstream defense genes, encoding, for example, plant defense hormone biosynthesis proteins, proteins involved in the production of reactive oxygen species, specialized metabolites, and reinforcement of the plant cell wall and PR proteins [48–51]. In *Arabidopsis thaliana*, wheat, maize, and rice, major-effect QRLs linked with resistance frequently harbor genes that encode proteins involved in pathogen perception or in downstream signaling, which in turn induce defense responses at various strengths [30, 49, 145, 146]. However, so far, not a single candidate allele of a gene from the identified QRLs for scab resistance has been functionally validated in apple.

Inoculation of resistant cultivars harboring different QRLs with individual pathogenic strains and races could benefit in gaining insights into the pathogen specificity of these QRLs. However, the initial QRL studies used mixtures of multiple different *V. inaequalis* strains and races in field [37] or greenhouse settings [38, 144], hence, intrinsically aiming at identification of major-effect QRLs that are effective against the tested pathogens [145]. More recently, inoculations with individual or a combination of known *V. inaequalis* strains belonging to different races enabled discrimination of the efficacy of individual QRLs toward specific strains [38, 47, 140, 144, 147]. QRLs that are effective against a broad range of pathogenic strains and races were discovered in cultivars ‘Discovery’ on LG2, -5, and -17; in ‘Prima’ on LG11; in ‘Fiesta’ on LG3, -11, and -17; in ‘Gala’ on LG11 and -17; and in TN10–8 on LG1, -2, -13, and -17 (Suppl. Table 2). In contrast, QRLs that are putatively effective against a very narrow selection of strains are present in ‘Prima’ on LG1 and -15, in TN10-8 on LG10, in ‘Fiesta’ on LG12, and in ‘Discovery’ on LG12, -13, and -15 [47, 144]. Interestingly, QRLs identified at specific positions on LG1, -2, -11, -15, and -17 were found in different genotypes, which could indicate the presence of common alleles for each of these QRLs across the different genotypes. Their identity by descent or state should be clarified. For example, the QRL on LG1 of TN10-8 is associated with the CH-Vf1–139 bp marker, which was found to be linked to the *Rvi17* resistance allele in ‘Antonovka’ APF22, a cultivar closely related to TN10-8 [148], as well as to the *Vhc1* resistance allele in ‘Honeycrisp’ [128]. However, TN10-8 plants are susceptible to *V. inaequalis* EU-NL24 [40], which does not correlate with the observation that *Rvi17* is linked with resistance to the same strains and races [148]. In general, it remains unclear how effective the identified QRLs are against various *V. inaequalis* strains and in different environments, particularly when integrated in different genetic backgrounds.

Understanding specific effects and stability of QRLs in various genotypes is crucial for their applicability in breeding. So far, only QRLs on LG 11 and 17 were explored in multiple progenies and under field and greenhouse conditions [37, 38, 47, 140, 144]. However, their validation in crosses with other apple cultivars is still required. In case markers can be identified as linked to these candidate broad-spectrum QRLs, introgression of these QRLs in other progenies with a different genetic background can be possible. Nevertheless, in order to promote the discovery of new stable QRLs in various genetic backgrounds, future QRL mapping endeavors should integrate larger multi-parent mapping populations with phenotypic evaluations based on a continuous scaling methodology [49].

## “-Omics” approaches provide insights into temporal and spatial processes underlying apple scab resistance

### Transcriptomics

To understand which plant responses play a role in defense against apple scab, several studies have compared transcriptomes of apple cultivars with different levels of resistance, either before or upon inoculation of leaves with *V. inaequalis* (Suppl. Tables 4, 5, and 6) [41, 42, 94, 149–156]. Transcriptomes of scab-susceptible cultivars ‘Gala’ [41, 42, 154, 155], ‘Golden Delicious’ [94, 150–152], and ‘Elstar’ [156], polygenic resistant ‘Président Roulin’ [41, 42], and *Rvi6*-harboring ‘Remo’ [41, 42, 94, 149–156], ‘Rewena’ [152], ‘Florina’ [150, 151], and ‘Gala’ transformed with the *HcrVf2* resistance allele of *Rvi6* [149, 154, 155] have been analyzed. These studies revealed two important aspects of the defense against apple scab. Firstly, susceptible and resistant genotypes show constitutive differences in expression of genes involved in various defense pathways. Secondly, inoculation with *V. inaequalis* additionally activates defense mechanisms in resistant and susceptible genotypes in a time-specific manner. However, as these studies show a high degree of variability in experimental conditions and plants inoculated with various *V. inaequalis* strains and races are compared at different time points using multiple techniques to measure gene expression, caution is warranted when drawing general conclusions about the transcriptional responses of different cultivars to *V. inaequalis*.

Transcriptome comparisons of *Rvi6*-based scab-resistant plants with plants lacking *Rvi6* under non-inoculated conditions show that the enhanced resistance of *Rvi6* genotypes is partially established constitutively, as *Rvi6* correlates with changes in expression of several genes (Suppl. Table 5). In the absence of infection, several genes involved in plant defense, ROS accumulation, and photosynthesis generally show increased expression in *Rvi6* genotypes compared with susceptible genotypes [41, 156], while genes involved in the biosynthesis of various primary and specialized metabolites, and other genes involved in ROS accumulation and photosynthesis show reduced expression [41, 156]. Constitutive differences in expression of a rather confined set of genes could, on the one hand, provide basal defense against potential pathogen infections and, on the other hand, may maintain high overall plant fitness as it might require only low amount of energy [157].

The constitutive activation of defense-related genes in *Rvi6* genotypes most likely involves only part of the plant defensive arsenal, as additional genes are differentially expressed upon *V. inaequalis* infection [155]. Transcriptomic analysis of transgenic and non-transgenic genotypes harboring the *HcrVf2* allele demonstrated that it induced defense response upon *V. inaequalis* inoculation based on various defense pathways (Suppl. Tables 5 and 6) [150, 152, 154–156]. Upon infection, *Rvi6* apple genotypes show an increased expression of various genes encoding receptor-like kinases, including the *HcrVf2* allele, as compared to susceptible genotypes, indicating an enhanced pathogen recognition capacity [150, 154, 155]. This is accompanied by a transcriptional upregulation of the salicylic acid (SA) pathway, characteristic for the response against biotrophic pathogens such as *V. inaequalis*, as well as the jasmonic acid and brassinosteroid pathways [154, 155]. These activated signaling cascades are followed by an increased expression of genes involved in ROS production and other defense-related processes [152, 154]. The enhanced defense mechanisms could explain the appearance of necrotic pits and inhibition of fungal growth on the leaf, which are attributed to the HR characteristic for some *Rvi6*-harboring scab-resistant genotypes [2, 93, 94]. In contrast, primary and specialized metabolism (e.g. lignification and flavonoid biosynthesis genes) can, depending on the genotype, either be up- or downregulated in *Rvi6* genotypes upon scab infection [152, 154, 155].

The timing of the defense response is critical in ensuring effective resistance against *V. inaequalis* infection, as has been demonstrated in comparisons between infected and non-infected plants at various time points post-inoculation, delineating distinct defense responses that occur either immediately or later upon infection [41, 42, 94, 149–152]. The most critical transcriptional reprogramming occurs within the first hours/days upon infection, i.e. referring to the early responses between 0 and 3 dpi, when the highest number of differentially expressed genes (DEGs) can be identified [155]. During this early response phase, resistant genotypes show a larger number of genes with enhanced expression in comparison to susceptible genotypes, although some genes that are involved in similar molecular mechanisms are differentially expressed in both genotypes (Suppl. Table 5). In contrast, in scab-susceptible cultivars, transcriptional upregulation of defense-related genes (e.g. encoding PR proteins, redox genes, etc.) is observed at later time points, e.g. at more than 5 dpi, thereby coinciding with a delayed or less effective recognition of the pathogen infection [152]. However, as defense responses of resistant and susceptible cultivars encompass similar processes that differ in timing of their enhancement, transcriptome studies of susceptible cultivars at various time points as compared with resistant cultivars could aid in identifying “the point of no return” for the effective timely response to the infection.

Transcriptome profiling has also been employed to unravel mechanism(s) underlying ontogenic resistance in ‘Golden Delicious’ leaves (Suppl. Tables 4 and 5) [94]. The enhanced resistance of older leaves correlates with a constitutive higher expression of genes involved in redox homeostasis, in production of defense-related metabolites, and in synthesis of cell wall components, callose, wax, and lignin precursors, such as phenolic compounds [94]. The latter strongly corroborates with microscopic observations suggesting that ontogenic scab resistance is attributed to the formation of physical barriers that inhibit acquisition of nutrients from host cells, in combination with accumulation of compounds directly toxic to the pathogen and enhanced abundance of redox enzymes putatively mediating the HR of the host [2, 93, 94]. Further transcriptomic studies of cultivars that exhibit distinct variation in the level of ontogenic resistance are required to confirm the underlying mechanisms [2, 71].

### Proteomics

Complementary to transcriptome profiling, quantitative and qualitative analyses of the apple proteome can offer additional insights into the contribution and specific localization of defense proteins acting against *V. inaequalis* [60, 158]. So far, two different strategies of protein extraction have been used to identify proteins that are potentially involved in the defense response: firstly, extraction from whole leaf tissue, and secondly, extraction from the intercellular apoplastic fluid [60, 158]. In particular the latter strategy could be relevant in the search for host proteins that directly interact with the fungus since *V. inaequalis* grows subcuticularly and lacks haustoria, and therefore does not directly harm living cells. In addition, the analysis of the apoplastic fluid might prove powerful in detecting low-abundant proteins that are critical for resistance, which may be much more difficult when using the whole leaf proteome due to biases toward the most abundant proteins [159].

Protein profiling using the two extraction methods on leaves of scab-susceptible ‘Golden Delicious’ and ‘Elstar’, and of *Rvi6* cultivars ‘Topaz’ and ‘Remo’, corroborates findings at the transcriptional level under non-infected conditions and during late infection responses, with the additional identification of proteins that are secreted into the apoplastic fluid (Suppl. Tables 6 and 7). In contrast to these conditions, for the early responses to infection, proteomics profiling results do not align with changes observed at the transcriptional level.

The studies revealed an important role of the apoplast as a first line of defense. More specifically, scab-resistant cultivars establish an apoplastic environment in the host tissue that is unfavorable for *V. inaequalis* colonization by constitutively secreting and accumulating increased amounts of defense proteins, including β-1,3-glucanase, chitinase, and thaumatin-like protein into the apoplastic fluid [158]. In addition, the complete leaf tissue of resistant cultivars shows a constitutively increased accumulation of several defense proteins, proteins involved in redox homeostasis, photosynthesis, and primary and specialized metabolism (e.g. glycolysis, phenylpropanoid biosynthesis), whereas other proteins involved in specific primary and specialized metabolism are decreased (e.g. glycolysis, phenylpropanoid biosynthesis) [60]. The defense mechanisms that are constitutively activated in resistant apple cultivars are also enhanced as a part of the later response upon *V. inaequalis* infection in resistant apple cultivars, whereas the amounts of some plant defense proteins, photosynthesis, and proteins involved in the biosynthesis of primary and specialized metabolites are reduced [60, 158]. Proteomics studies of susceptible cultivars revealed that similar defense mechanisms are also enhanced, although only at a later colonization stage, with an increased secretion and accumulation of defense proteins in the apoplastic fluid to levels similar as in resistant cultivars [158]. The activity of both plant as well as pathogen proteins secreted in the apoplastic fluid can be affected by the apoplastic pH [2, 160, 161], impacting apple scab development and resistance. Additional apoplastic proteomics studies, immediately upon infection, by characterizing the leaf protein profile at earlier time points are key to validate whether pathogen recognition is effectively enhanced in scab-resistant cultivars, and which pathways are actually involved in the defense response [162].

Another advantage of proteomics analyses is the possibility to detect allele-specific protein variants, without the need of prior knowledge of the genome sequence [163]. Identification and functional characterization of allele-specific proteins could critically improve our understanding of scab resistance, as it has been demonstrated that the open reading frame sequence of genes involved in defense response varies in different apple genotypes [125, 132, 164]. Upon infection of apple plants, different isoforms of several metabolism proteins (e.g. phosphoglycerate kinase) were either found to show a different cellular localization, while others involved in plant defense (e.g. Mal d 1) exhibit a differential accumulation between resistant and susceptible cultivars [60], with a tendency toward higher accumulation of one isoform in the resistant cultivar. However, the specific role of discovered protein isoforms and their contribution to scab resistance/susceptibility is yet unknown.

### Metabolomics

Plant metabolites have repeatedly been suggested to play a major role in the defense response of apple against *V. inaequalis* [10] and include phenolic compounds, plant hormones, organic acids, and sugars. So far, profiling of these metabolites in apple has been performed on different tissues, including leaves, fruit skin, and pulp, with a main focus on phenolic compounds [165–172] (Suppl. Table 8).

Several metabolomics studies have revealed that apple leaf tissue infected with *V. inaequalis* contains increased amounts of total phenolic compounds (TPCs), as compared to healthy tissue [166, 168, 170, 173]. More specifically, the amounts of TPCs in naturally infected leaves of orchard-grown susceptible cultivars ‘Golden Delicious’ and ‘Jonagold’ were on average up to 140% and 20% higher, respectively, than in healthy leaves of the same tree [166, 168]. Although these results might have been affected by environmental conditions and the presence of other pathogens, polyphenolic compounds most likely contribute to the defense response, as individual phenolics belonging to e.g. hydroxycinnamic acids, dihydrochalcones, and flavanols can have a direct *in vitro* or *in vivo* inhibitory effect on the growth of *V. inaequalis* [2, 174, 175]. Furthermore, the pathogen growth-inhibiting role of phenolic compounds in the leaves of the scab-resistant cultivar ‘Sir Prize’ was demonstrated via chemical inhibition of phenylalanine-ammonia-lyase (PAL), one of the key enzymes in the biosynthesis of phenolic compounds [176]. PAL inhibition in this cultivar resulted in larger scab lesions compared with lesions observed on mock-treated leaves. Nevertheless, comparative analysis of different cultivars revealed that higher amounts of TPCs do not consistently correlate with increased scab resistance, and therefore additional research of specific phenolic compounds is required to understand which specific compounds contribute to the increased resistance [170–173].

Studies of specific phenolic compounds suggest that their increased accumulation in symptomless leaves after inoculation in the field reinforces resistance to *V. inaequalis*. Upon infection, increased amounts of hydroxycinnamic acids (chlorogenic acid, ferulic acid, and coumaric acids), dihydrochalcones (phloridzin and phloretin), flavonols (isoquercitrin, quercitrin, hiperoside, and rutin), ‘phenol 173’ (phenol with retention time 173.38 min), gallic acid, and flavanols (catechins and proanthocyanidins) were measured [165, 168, 172, 177–179]. Among these compounds, some have shown strong potential to inhibit *V. inaequalis* colonization. Firstly, coumaric acids and chlorogenic acid can inhibit the growth and sporulation of *V. inaequalis in vitro* and on *in vivo* apple leaves when injected into apple shoots. However, they can readily be broken down by polyphenol oxidases, which can reduce their inhibiting role [2, 174, 180–182]. Secondly, phloretin and phloridzin were also suggested to be strong candidates involved in the inhibition of *V. inaequalis*, although inconsistencies related to their antimicrobial activity exist [2]. *In vitro* studies revealed that phloretin has a high intrinsic potential to inhibit *V. inaequalis* growth [2, 183, 184]*,* although data on leaf homogenates suggest that the compound is inhibitory only at concentrations that are rarely reached in leaves [185]. Phloridzin, one of the most abundant phenolic compounds in apple leaves, can be metabolized by the fungus and can even increase mycelial growth of *V. inaequalis in vitro* [2, 183, 186]. Since the inhibition of oxidation can enhance pathogen growth, the oxidation products, i.e. *o*-quinones, of either phloretin or phloridzin, rather than the compounds themselves, might have a major role in resistance, as observed *in vitro* [2, 184, 185, 187]. Thirdly, the amounts of the flavanols catechin, epicatechin, and procyanidins are generally higher in infected leaves of resistant cultivars, as compared with those of susceptible cultivars [167]. These flavanols accumulate locally in response to *V. inaequalis* infection, as observed by staining lesions with *p*-dimethylaminocinnamaldehyde (DMACA), suggesting a putative role in inhibiting fungal infection [176, 188]. However, although the majority of the abovementioned studies proposed strong involvement of flavanols in the resistance to apple scab, no correlation could be found between the amount of flavanols and *V. inaequalis* leaf resistance in the progenies of crosses between *Rvi6* cultivar ‘Florina’ and the scab-susceptible cultivars ‘Spartan’ and ‘Glockenapfel’ [189]. In addition, the amount of flavanols in the leaves of *Rvi2*, *-11*, and *-12* cultivars did not correlate with increased scab resistance in comparison to susceptible cultivars [2].

Other plant metabolites with a putative role in apple scab resistance comprise biphenyls and dibenzofurans [131, 190, 191]. An increased accumulation of the biphenyls aucuparin and noraucuparin, and the dibenzofuran, eriobofuran, was observed in *in vitro* cell suspension cultures of the *Rvi6* cultivar ‘Florina’ upon treatment with *V. inaequalis* elicitors compared to the susceptible cultivar ‘Vista Bella’ [131]. Further *in vitro* studies revealed that all these compounds inhibit *V. inaequalis* spore germination, indicating direct antifungal activity [131]. Among these compounds, only biphenyls accumulate *in vivo* in stem tissues of *V. inaequalis* inoculated ‘Florina’, whereas they all remain undetected in the leaves [190].

It is important to note that most metabolomics studies were performed on orchard-grown apple trees, and thus may be biased due to environmental conditions and the lack of data on exact infection time points. The specific role of phenolic compounds in resistance to apple scab should therefore be studied in a controlled-environment and with artificial inoculation. Similarly to proteomics analyses, valuable information on important metabolites could be obtained by separately analyzing apoplastic and symplastic metabolites.

### Integrating “-omics” approaches on the *Venturia inaequalis*–*Malus* × *domestica* interaction

**Figure 5:**
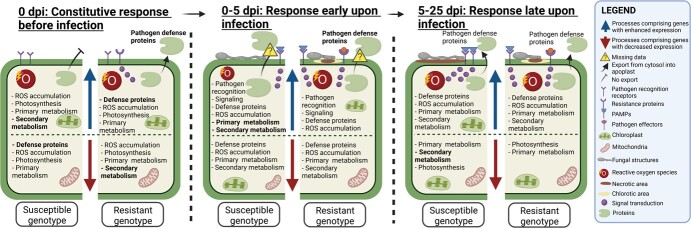
Schematic representation of constitutive defense responses (0 days post-inoculation (dpi)) and early (0–5 dpi) and late (5–25 dpi) defense responses upon *V. inaequalis* infection in apple plants compared to non-infected plants. Processes comprising genes with enhanced (blue arrows) and decreased (red arrows) expression encompass various numbers of different genes associated with these processes; bold text indicates processes unique for each condition tested. The scheme is based on the transcriptomics and proteomics data reviewed in section 4. Specific defense proteins are constitutively exported from the cytosol into the apoplast in resistant genotypes, while this occurs from 5 to 25 dpi in susceptible genotypes (black arrows). Such data are missing for defense responses directly upon infection between 0 and 5 dpi (yellow signs with a question mark). The infection progresses more in the case of susceptible genotypes (gray fungal structures) and induces extended necrosis (brown area underneath the fungal structures) in comparison to the resistant genotypes, leading to reduced necrosis and chlorosis (yellow area surrounding necrotic area). Pathogen recognition receptors (PRRs; rectangular-shaped) and resistance proteins (R; round-shaped), which recognize pathogen/damage-associated molecular patterns (P/DAMPs, blue triangles) and pathogen effectors (orange circles), respectively, and trigger signal transduction (three purple circles) upon infection.

To conclude, linking transcriptomics, proteomics, and metabolomics studies on *V. inaequalis*–*M.* × *domestica* interaction could fill current knowledge gaps and provide insights into the signaling pathways and defense responses underlying qualitative and quantitative scab resistance (Suppl. Table 5 and Fig. 5). However, the reported studies are based on different experimental setups that include different environmental conditions and do not integrate different “-omics” approaches in a systematic way. In addition, many defense mechanisms as conferred by *Rvi* genes other than *Rvi6* and other QRLs have, to the best of our knowledge, not been characterized yet. Filling the existing knowledge gaps associated with scab resistance mechanisms and specific gene-to-trait associations will require in-depth comparisons between a broader range of resistant and susceptible apple genotypes inoculated with a variety of *V. inaequalis* strains, using state-of-the-art approaches, including advanced phenotyping, RNAseq-based transcriptomics, and mass spectrometry-based proteomics and metabolomics.

## Implementation of enhanced scab resistance into apple cultivars

The ultimate goal of apple scab research is to develop apple cultivars with prolonged enhanced resistance [30, 146, 192]. Quantitative resistance based on multiple minor-effect alleles involved in various defense mechanisms alongside pathogen recognition has been frequently suggested as the best strategy for obtaining enhanced resistance against apple scab [28, 37–39, 47, 144, 193]. Several QRLs have been found to harbor alleles that cumulatively lead to an increased (yet incomplete) resistance against *V. inaequalis*, and most likely many more yet unidentified QRLs are involved [37–39, 47, 144]. As the underlying alleles have not been characterized yet, the identification of genes with different functionalities would be highly relevant for the targeted build-up of enhanced resistance. However, this will require additional population and functional genetic studies that focus on identifying combinations of alleles that evoke maximum fitness cost for the pathogen [9]. Ideally, enhanced resistance against apple scab may also be obtained by combining QRLs with multiple types of *Rvi* genes. However, the exact number of such genes sufficient to establish durable resistance is unknown. As long as little is known about the exact mechanistic contribution of specific *Rvi* genes and their interaction with other genes, apple breeders are confined to randomly combining resistance sources, i.e. QRLs and *Rvi* loci, into novel genotypes to eventually identify specific allelic combinations that confer enhanced scab resistance. Such untargeted conventional breeding approach requires several breeding cycles and large progenies (i.e. with repetitive rounds of clonal phenotyping), and thus is laborious and time consuming. In the following paragraphs, we pinpoint the progress made toward improved breeding for enhanced scab resistance.

### Conventional breeding for durable scab resistance

#### Resistance markers

While in the past, apple scab breeding mainly focused on the introgression of individual *Rvi* genes, with genotype selection mainly occurring via distinct phenotypic reactions during artificial or natural inoculation assays, nowadays, marker-assisted selection (MAS) using genetic markers associated with disease resistance has been widely used (Suppl. Table 9). Initially, the focus was mainly on the *Rvi6* locus, though currently increasingly more markers are developed that are linked with various resistance loci (Suppl. Table 9). The early markers were often located ~10 cM from the actual gene of interest, while several recent *Rvi*-associated markers have been identified as less than 1 cM from the gene. The first scab resistance markers in apple were based on random amplification of polymorphic DNA (RAPD) and were for example linked to *Rvi6* and *Rvi11* [194–198]. Most of these RAPD markers have been converted into sequence-characterized amplified region markers and cleaved amplified polymorphic sequences [199, 200] to conveniently detect the gene-of-interest in practice. Later on, amplified fragment length polymorphism (AFLP) markers tightly linked to various *Rvi* loci were mapped [201]. In parallel, simple sequence repeat markers (SSRs) were used to genotype apple populations, and several of them were found to be linked to major *Rvi* loci, as for example for *Rvi6*, for which two linked SSR markers were identified, i.e. CH-Vf1 and CH-Vf2 [202–205]. However, the most broadly used markers today are single-nucleotide polymorphism markers (SNP), which were mainly discovered in biparental populations across the genome and have been linked to the majority of scab resistance loci [206–209]. The use of SNP microarrays, covering a large part of the apple genome including scab resistance loci, enables convenient high-throughput SNP genotyping with an increased resolution through the increased saturation [106, 210–214]. The continuous development of SNP arrays detecting a larger number of SNPs as well as new sequencing-based SNP genotyping approaches now allows for linkage-based mapping of both monogenic and polygenic scab resistance from various germplasm resistance sources.

#### Pyramiding *Rvi* loci and QRLs

It has been regularly suggested that the durability of qualitative resistance in apple cultivars can be improved by the pyramiding of effective *Rvi* genes and QRLs [120, 129, 130]. However, though studies of gene pyramiding for scab resistance are rather limited, they demonstrated that even individually effective *Rvi* genes can be overcome when pyramided and that such approach does not guarantee durable resistance [44, 45]. The careful selection of *Rvi* genes for gene pyramiding is crucial to enhance durable resistance, as some *Rvi*-based resistance mechanisms show less tendency in being overcome by the fungal pathogen than others. *Rvi11* and *Rvi4* resistance (in GMAL2473), for instance, have not been overcome so far, and also *Rvi5*, *Rvi12*, and *Rvi14* resistance have rarely been linked to resistance breakdown, most likely because these genes have been introgressed in apple cultivars that are not commonly adopted or only used in local production areas, or were exposed to a low infection pressure [43]. Successful pyramiding of scab resistance genes acting against a broad spectrum of *V. inaequalis* strains has been demonstrated in experimental hybridizations of seedlings from a cross between ‘Regia’ and ‘Ariwa’ that carry different combinations of *Rvi2* and *Rvi4* from ‘Regia’ and *Rvi6* from ‘Ariwa’ [44]. However, *Rvi* loci pyramiding alone is insufficient for providing sustainable resistance, as in ‘Regia’ the resistance has been overcome by virulent *V. inaequalis* strains, despite the presence of pyramided *Rvi2* and *Rvi4* [102].

Resistance combined by various QRLs in a genotype has been eroded over time. QRL pyramiding was demonstrated in a cross between genotype TN10-8 harboring the T1 QRL, and ‘Fiesta’ harboring the F11 and F17 QRLs [46]. Although incomplete, the highest level of disease resistance was observed in plants carrying all three QRLs against all 10 isolates tested, followed by plants carrying either T1 or a combination of F11 and F17 (T1–F11 or T1–F17 combinations were not tested), while plants without any QRLs showed the highest level of sporulation [46]. Despite the beneficial effect of QRL pyramiding on scab resistance, erosion of QRLs can still occur within a decade, even when multiple QRLs are stacked in a single cultivar, as demonstrated for cultivars carrying a combination of F11, F17, and T1, or F11 and F17 [30, 99–101]. Strikingly, scab resistance eroded faster in areas where cultivars with one individual or multiple QRLs were present already before the experiment, indicating that evolutionary adaptation to the host’s resistance mechanisms occurs gradually, which could affect the usefulness of pyramids [101]. Nevertheless, little is known about the extent and rate of erosion of quantitative scab resistance and the genetic elements that influence this, as well as if certain pyramided QRLs can enhance durability of vertical resistance controlled by an *Rvi* gene. Regional screening of *V. inaequalis* races, as exemplified for *Rvi* loci in the Vinquest project [43], could be applied to the known QRLs and would aid in preventing rapid breakdowns of the resistance in novel cultivars harboring pyramided resistance QRLs.

In addition, the existing literature on the pyramiding of specific genes-of-interest does not provide evidence whether pyramiding of specific resistance genes is affected by the environmental conditions and if it may confer specific adverse effects on other relevant traits, such as fruit quality or fruit productivity, due to linkage drag. These aspects will determine the success of novel scab-resistant cultivars, and hence should be further investigated.

### Polyploidy induction

Like in many other crop species, polyploidy in apple is often related to higher adaptability to stress [78, 215–220], though scab susceptibility of polyploid cultivars has rarely been studied [78, 79, 221]. Tetraploid lines of scab-susceptible ‘Gala’ and the *Rvi6*-harboring ‘Makali’ [78, 79] and ‘Free Redstar’ [221] show reduced *V. inaequalis* infection compared with their diploid counterparts. The study in ‘Free Redstar’ indicated that this enhanced scab resistance could be attributed to ploidy-related dosage effects of alleles involved in pathogen defense (e.g. *CERK1*, *PR1*, *WRKY29*, *CDPK*, and *MPK4*) or in pathogen recognition (i.e. *Rvi6*) upon infection with *V. inaequalis* [221], similarly as was observed for *Rvi6* homozygous plants as compared to their heterozygous counterparts [222]. Nevertheless, in terms of durability, polyploidy may be advantageous compared to diploid plants, as the enhanced dosage of effective alleles of genes involved in downstream defense responses could result particularly in enhanced effects of versatile minor-effect alleles. Therefore, further research is required to validate broad-scale applicability of polyploidy for increasing scab resistance in apple genotypes. However, implementation of polyploidy into existing breeding programs would require adaptations of breeding schemes that are currently predominantly adapted to utilizing diploid accessions. This would encompass generation of autotetraploid genotypes for crossing with diploids to generate triploid progeny [223, 224].

### Transgenesis and cisgenesis

Apart from classical breeding strategies, reduced scab susceptibility can be achieved via genetic transformation using trans- or cisgenesis [225]. In the case of transgenesis, genes from distant species encoding either proteins directly toxic to the pathogen or with a role in defense signaling (Suppl. Table 3) have been introduced in apple [226–232]. For example, constitutive heterologous expression of exochitinase *Nag70* or endochitinase *Ech42* from *Trichoderma* reduced the susceptibility of ‘Marshall McIntosh’ and ‘Royal Gala’ against apple scab, resulting from an inhibitory effect on fungal growth through random cleavage of chitin, a component of the fungal cell wall [229]. For some of these transgenic lines resistance levels were equal to that of the *Rvi6* cultivar ‘Liberty’ [228, 233, 234]. In addition, plants containing both *Nag70* and *Ech42* showed a reduced susceptibility against various *V. inaequalis* races as compared to non-transgenic control plants [229, 231, 233, 235]. However, in none of the abovementioned cases complete absence of sporulation was achieved. Other genes encoding products that are directly toxic to the pathogen showed promising effects on apple scab resistance as well. For example, scab-susceptible ‘Galaxy’ transformed with puroindoline-B (*pinB*) from wheat, which enables formation of ion channels in lipid membranes [236], resulted in decreased susceptibility to race (6), but had no effect on race (1). Similarly, *pinB*-transformed ‘Ariane’ showed reduced susceptibility to race (6), while race (1) was not tested [231]. Transformation of ‘Jonagold’ with the antimicrobial protein Ace (*AMP-1*) from onion resulted in reduced scab susceptibility of some lines [226, 232]. Heterologous expression of *Leaf colour* (*Lc*), controlling anthocyanin biosynthesis from maize, in ‘Holsteiner Cox’ and ‘Galaxy’ resulted in reduced susceptibility to scab, most likely through increased flavonol contents accompanied by the presence of HR lesions. However, overexpression of *Lc* additionally resulted in antagonistic pleiotropic effects including reduced trichome development, reduced shoot diameter, and curled discolored leaves. In all transgenic lines mentioned above, the recombinant cassette was driven by the constitutive CaMV 35S promoter, which in combination with selected transgenes could affect commercially relevant traits and lead to reduced vigor, fruit yield, and quality [230, 234, 237, 238]. These undesired side-effects may putatively be avoided by driving the transgenes by native *Rvi* promoter [239].

In case of cisgenesis, genes from the same species or from sexually cross-compatible species are transformed into the desired plant. For *cis* introduction of scab resistance, *Rvi6* and *Rvi4* resistance alleles *HcrVf2* and *Vr2-C* under control of their native promoters originating from *M. floribunda* 821 and GMAL2473, respectively, have been introduced into the susceptible apple cultivars ‘Gala’, ‘McIntosh’, and ‘Elstar’ and substantially reduced apple scab symptoms to a level that was comparable to resistant cultivars [123, 129, 240–242]. Similarly, overexpression of *nonexpressor of PR1* (*MpNPR1*), an SA-dependent activator of PR genes from ‘Jonathan’, in Galaxy resulted in reduced susceptibility to scab [243].

An important factor influencing the effectiveness of a transgene to modulate apple scab susceptibility scab is the promotor sequence used. In transgenic ‘Elstar’ and ‘Gala’ expressing *HcrVf2*, for example, the length of the promoter appears to be important for *HcrVf2* expression and the level of resistance [23, 133, 239]. While a native *HcrVf2* promoter shorter than 115 bp does not confer resistance in cisgenic plants due to lack of *HcrVf2* expression [133], longer promotors generally resulted in a sufficient, but highly variable expression of the resistance gene [23, 129, 133, 239]. Altogether, the integration of cisgenes, removal of transgene cassette, and stable integration and expression of the gene under control of a native promoter could improve overall acceptance by consumers, as such approach results in plants that metabolically do not differ from wild-type plants [244, 245].

### Genome editing and targeted mutagenesis

The availability of the apple genome sequence and particularly the functional annotation of genes in combination with novel genome editing tools enables targeted mutations in specific genomic sites of interest [246]. CRISPR/Cas9 shows the highest potential to modify the apple genome, as it confers broad flexibility in gene targeting with reported transformation efficiencies of up to 24% or 40% for ‘Galaxy’ and M.26, respectively [246, 247].

Genome editing is yet to be tested to improve scab resistance and will depend on the future discovery of candidate genes and their resistance alleles that can reduce the susceptibility of apple to *V. inaequalis* infection. One of the valid approaches could be to target susceptibility (*S*) genes, i.e. negative regulators of plant immunity. The expression of these genes can be induced by the pathogen effector, as for instance by specific binding to the promoter of the gene locus [248], or the pathogen effector can stimulate protein activity through direct interactions and as such stabilize an S protein [249]. By introducing targeted mutations in the effector-binding site of the promoter region or in the coding sequence, the recognition and binding of the effector molecule is hindered, leading to a reduced or depleted expression of *S* genes, or reduced activity of the encoded protein [248, 250, 251]. Although knowledge on scab-related *S* genes is lacking, several *S* genes have already been found to play a role in apple’s resistance to other diseases such as fire blight, *Alternaria* blotch, and powdery mildew [252–255].

In addition to *S* genes, *Rvi* loci and QRLs that contribute to resistance to *V. inaequalis* might carry suboptimal alleles [251, 256]. Once the suboptimal alleles have been identified in various elite apple cultivars, these alleles can be edited toward the optimal allele sequence known for resistance to *V. inaequalis*, as exemplified earlier [257].

However, genome editing using CRISPR/Cas9 may be subjected to random/off-target effects in the genome, infliction of different mutations, chimerism [247], as well as recalcitrance of specific genotypes to genetic transformation or regeneration [251, 254]. As only a few apple genotypes have been tested so far and since modified plants were not yet grown in the field, the potential effects of the editing on economically valuable fruit quality characteristics is yet to be researched.

## Conclusions on breeding for enhanced scab resistance

Conventional breeding strategies using molecular markers in combination with genome editing can significantly accelerate the introgression of resistance alleles in existing commercial cultivars [240, 241, 258, 259]. However, although the adoption of scab-resistant cultivars developed via the described approaches would provide a big step forward for sustainable apple production, the low degree of acceptance by the public, the restrictive and changing legislation on GMOs in some regions, and putative off-target effects are still a major obstacle hindering their broad-scale implementation. Utilizing modern speed apple breeding approaches, based on accurate modifications of apple genomes, these obstacles may possibly be overcome and enable implementation of enhanced scab resistance in commercial apple production.

## Supplementary Material

Web_Material_uhae002

## Data Availability

All data are included in this manuscript.
