# Supplementary material for: Phenotyping, genetics, and “-omics” approaches to unravel and introgress enhanced resistance against apple scab (Venturia inaequalis) in apple cultivars (Malus × domestica)
Source: Hortic Res. 2024 Jan 10;11(2):uhae002. doi: 10.1093/hr/uhae002 (PMC10873587; doi:10.1093/hr/uhae002)
Supplement: Web_Material_uhae002 [file web_material_uhae002.zip › REVIEWED Supplementary table 1 Major R genes.doc]

Supplementary table 1 Major *R* loci in apple against *V. inaequalis*, *Avr* loci, races lacking the respective *Avr* loci, and resistance reaction after the infection, updated from^1^; * Symptoms range: no lesions, chlorotic lesions, necrotic lesions, i.e. indicating hypersensitive response, stellate necrosis and sporulation; old – historical nomenclature of apple resistance loci; new – nomenclature according to Bus et al.^1^ ; LG – linkage group.

| ***Malus*** | | | | | | ***Venturia inaequalis*** | | |
| --- | --- | --- | --- | --- | --- | --- | --- | --- |
| Symptoms | Host accession | Host number | Resistance locus (old) | Resistance locus (new) | Location (LG) | Avirulence locus | Race | Ref. |
| Sporulation | 'Royal Gala' | H0 | / | / | / | / | 0 |  |
| Chlorosis | 'Priscilla' | H6 | *Vf* | *Rvi6* | 1 | *AvrRvi6* | 6 | ^2,3^ |
| Chlorosis | 'Hansen’s baccata #2' | H12 | *Vb* | *Rvi12* | 12 | *AvrRvi12* | 12 | ^4,5^ |
| Chlorosis | 'Dülmener Rosenapfel' | H14 | *Vdr1* | *Rvi14* | 6 | *AvrRvi14* | 14 | ^6^ |
| Chlorosis | Antonovka APF22 | H17 | *Va1* | *Rvi17* | 1 | *AvrRvi17* | 17 | ^7^ |
| Stellate necrosis/chlorosis | A722–7 | H11 | *Vbj* | *Rvi11* | 2 | *AvrRvi11* | 11 | ^8^ |
| Stellate necrosis | TSR34T15 | H2 | *Vh2* | *Rvi2* | 2 | *AvrRvi2* | 2 | ^4^ |
| Stellate necrosis | 'Geneva' | H3 | *Vh3* | *Rvi3* | 4 | *AvrRvi3* | 3 | ^9^ |
| Stellate necrosis | B45 | H8 | *Vh8* | *Rvi8* | 2 | *AvrRvi8* | 8 | ^10^ |
| Stellate necrosis | K2 | H9 | *Vdg* | *Rvi9* | 2 | *AvrRvi9* | 9 | ^11^ |
| Stellate necrosis | 'Durello di Forli' | H13 | *Vd* | *Rvi13* | 10 | *AvrRvi13* | 13 | ^12^ |
| Necrosis | 'Golden Delicious' | H1 | *Vg* | *Rvi1* | 12 | *AvrRvi1* | 1 | ^13^ |
| / | R12740-7A (russian seedling) | H19 | / | *Rvi19* | 1 | *AvrRvi19* | 19 | ^14^ |
| Hypersensitive response | TSR33T239 | H4 | *Vh4 = Vx = Vr1* | *Rvi4* | 2 | *AvrRvi4* | 4 | ^4^ |
| Hypersensitive response | 9-AR2T196 | H5 | *Vm* | *Rvi5* | 17 | *AvrRvi5* | 5 | ^15^ |
| Hypersensitive response | *Malus* x *floribunda* 821 | H7 | *Vfh* | *Rvi7* | 8 | *AvrRvi7* | 7 | ^16^ |
| Hypersensitive response | A723–6 | H10 | *Va* | *Rvi10* | 1 | *AvrRvi10* | 10 | ^17^ |
| Hypersensitive response* | GMAL2473 | H15 | *Vr2* | *Rvi15* | 2 | *AvrRvi15* | 15 | ^18^ |
| Hypersensitive response | MIS op 93.051 G07–098 | H16 | *Vmis* | *Rvi16* | 3 | *AvrRvi16* | 16 | ^19^ |
| Hypersensitive response | 1980-015-025 | H18 | *V25* | *Rvi18* | 11 | *AvrRvi18* | 18 | ^20^ |

**Rvi15* was identified as identical to *Rvi4*. The latter locus/gene is considered the reference locus/gene^21^.

1.	Bus, V. G. M., Rikkerink, E. H. A., Caffier, V., Durel, C.-E. & Plummer, K. M. Revision of the nomenclature of the differential host-pathogen interactions of *Venturia inaequalis* and *Malus*. *Annu. Rev. Phytopathol.* **49**, 391–413 (2011).

2.	Parisi, L., Lespinasse, Y., Guillaumes, J. & Krüger, J. A new race of *Venturia inaequalis* virulent to apples with resistance due to the *Vf* gene. *Phytopathology* **83**, 533–537 (1993).

3.	Patocchi, a., Gianfranceschi, L. & Gessler, C. Towards the map-based cloning of *Vf* : fine and physical mapping of the *Vf* Region. *TAG Theor. Appl. Genet.* **99**, 1012–1017 (1999).

4.	Bus, V. G. M. *et al.* The *Vh2* and *Vh4* scab resistance genes in two differential hosts derived from Russian apple R12740-7A map to the same linkage group of apple. *Mol. Breed.* **15**, 103–116 (2005).

5.	Padmarasu, S. *et al.* Fine-mapping of the apple scab resistance locus *Rvi12* (*Vb*) derived from ‘Hansen’s baccata #2’. *Mol. Breed.* **34**, 2119–2129 (2014).

6.	Soufflet-Freslon, V., Gianfranceschi, L., Patocchi, A. & Durel, C.-E. Inheritance studies of apple scab resistance and identification of *Rvi14*, a new major gene that acts together with other broad-spectrum QTL. *Genome* **51**, 657–67 (2008).

7.	Dunemann, F. & Egerer, J. A major resistance gene from Russian apple ‘Antonovka’ conferring field immunity against apple scab is closely linked to the *Vf* locus. *Tree Genet. Genomes* **6**, 627–633 (2010).

8.	Gygax, M. *et al.* Molecular markers linked to the apple scab resistance gene *Vbj* derived from *Malus baccata* jackii. *Theor. Appl. Genet.* **109**, 1702–9 (2004).

9.	Bastiaanse, H. *et al.* Scab resistance in ‘Geneva’ apple is conditioned by a resistance gene cluster with complex genetic control. *Mol. Plant Pathol.* **17**, 159–172 (2016).

10.	Bus, V. G. M. *et al.* The *Vh8* locus of a new gene-for-gene interaction between *Venturia inaequalis* and the wild apple *Malus sieversii* is closely linked to the *Vh2* locus in *Malus pumila* R12740-7A. *New Phytol.* **166**, 1035–1049 (2005).

11.	Broggini, G. A. L. *et al.* Genetic mapping of 14 avirulence genes in an EU-B04 × 1639 progeny of *Venturia inaequalis*. *Fungal Genet. Biol.* **48**, 166–176 (2011).

12.	Tartarini, S. *et al.* Characterisation and genetic mapping of a major scab resistance gene from the old Italian apple cultivar ‘Durello di Forli’. *Acta Hortic.* **663**, 129–134 (2004).

13.	Cova, V. *et al.* High-resolution genetic and physical map of the *Rvi1* (*Vg*) apple scab resistance locus. *Mol. Breed.* **35**, (2015).

14.	Gardiner, S. E. *et al.* Genetic mapping of novel loci for resistance to European canker and apple scab. in *9th International Rosaceae Genomics Conference* 36 (2018).

15.	Patocchi, A. *et al.* Identification by genome scanning approach (GSA) of a microsatellite tightly associated with the apple scab resistance gene *Vm*. *Genome* **48**, 630–636 (2005).

16.	Shay, J. R. & Hough, L. F. Evaluation of apple scab resistance in selections of *Malus*. *Am. J. Bot.* **39**, 288–297 (1952).

17.	Hemmat, M., Brown, S. K., Aldwinckle, H. S., Mehlenbacher, S. A. & Weeden, N. F. Identification and mapping of markers for resistance to apple scab from ‘Antonovka’ and ‘Hansen’s baccata #2’. *Acta Hortic.* **622**, 153–161 (2003).

18.	Patocchi, A., Bigler, B., Koller, B., Kellerhals, M. & Gessler, C. *Vr2*: A new apple scab resistance gene. *Theor. Appl. Genet.* **109**, 1087–1092 (2004).

19.	Bus, V. G. M. *et al.* Genome mapping of an apple scab, a powdery mildew and a woolly apple aphid resistance gene from open-pollinated mildew immune selection. *Tree Genet. Genomes* **6**, 477–487 (2010).

20.	Soriano, J. M. *et al.* Fine mapping of the gene *Rvi18* (*V25*) for broad-spectrum resistance to apple scab, and development of a linked SSR marker suitable for marker-assisted breeding. *Mol. Breed.* **34**, 2021–2032 (2014).

21.	Peil, A. *et al.* *Rvi4* and *Rvi15* are the same apple scab resistance genes. *Mol. Breed.* **43**, 74 (2023).
