# Supplementary material for: Phenotyping, genetics, and “-omics” approaches to unravel and introgress enhanced resistance against apple scab (Venturia inaequalis) in apple cultivars (Malus × domestica)
Source: Hortic Res. 2024 Jan 10;11(2):uhae002. doi: 10.1093/hr/uhae002 (PMC10873587; doi:10.1093/hr/uhae002)
Supplement: Web_Material_uhae002 [file web_material_uhae002.zip › REVIEWED Supplementary table 2 QRLs.doc]

Supplementary table 2 Quantitative resistance loci (QRL) identified in apple against *V. inaequalis*. LG – linkage group

| **Location** | **Marker** | **Cross parents and favourable allele carrier**** | ***V. inaequalis* strain** | **Variance in phenotype explained by the QRL (%)** | **Total variance genetically explained (%)** | **Reference** | **Remarks explaining the asterisk (*) in each line of the table.** |
| --- | --- | --- | --- | --- | --- | --- | --- |
| LG1 | CH-Vf1 | ‘Prima'** × 'Fiesta’ | 302 | 16.0, 17.8* | 70 | ^1^ | Explained variance for severity and sporulation class^2,3^ |
|  |  | ‘Discovery’ × ‘TN10-8’** | EU-B04 | 25.6 | 61 | ^4^ |  |
|  |  |  | EU-D42 | 13.7 | 13 |  |  |
|  |  |  | EU-NL24 | 4.1 | 38 |  |  |
|  |  |  | Gd-CH | 12.7 | 27 |  |  |
|  | CH-Vf1 | TN10-8** × Fiesta | EU-B04 | 69.9 | 81.9 | ^5^ |  |
|  |  |  | 09BCZ014 (2557) | 10.7 | 47.5 |  |  |
|  | PGM-1 | ‘Discovery’ × ‘TN10-8’** | MLI | 51.1 | 48 | ^4^ |  |
| LG2 | CH05e03 | ‘Discovery’** × ‘TN10-8’ | EU-B04 | 9.5 | 61 | ^4^ |  |
|  |  | ‘Discovery’** × ‘TN10-8’** | 104 | 8.4 | 31 |  |  |
|  |  | ‘Discovery’ × ‘TN10-8’** | 302 | 25.8 | 38 |  |  |
|  |  | ‘Discovery’** × ‘TN10-8’ | EU-NL24 | 25.7 | 38 |  |  |
|  | CH02c06 | ‘Discovery’ × ‘TN10-8’** | MLI | 5.1 | 48 |  |  |
|  | E33M49-15 | ‘Discovery’** × ‘TN10-8’** | EU-D42 | 13.0 | 13 |  |  |
| LG3 |  | TN10-8 × Fiesta** | EU-B04 | 5.5 | 65.1 | ^5^ |  |
|  |  |  | 09BCZ014 (2557) | 5.3 | 47.5 |  |  |
| LG5 | CH04e03 | ‘Discovery’** × ‘TN10-8’ | EU-B04 | 20.8 | 61 | ^4^ |  |
|  |  | ‘Discovery’** × ‘TN10-8’** | MLI | 12.5 | 48 |  |  |
| LG6 | HB09-SSR_DR | ‘Gala’ × ‘Dülmener Rosenapfel’** | Five isolates* | 12.3 | 54.8 | ^6^ | Isolates EU-B04, EU-NL19,EU-D42, EU-NL24 and 1066 |
|  |  | ‘Fiesta’** × ‘Discovery’ | Multiple strains | 5 | 55* | ^7^ |  |
| LG7 |  | ‘Fiesta’** × ‘Discovery’ | Multiple strains | 4 | 55* | ^7^ |  |
| LG10 |  | ‘Fiesta’** × ‘Discovery’ | Multiple strains | 6 | 55* | ^7^ |  |
|  |  | TN10-8** × Fiesta | 09BCZ014 (2557) | 3.5 | 47.5 | ^5^ |  |
| LG11 | CH04h02z, OPA-09-0600 | ‘Prima'** × 'Fiesta’** | 302 | 18.7, 22.6* | 70 | ^1^ | Explained variance for severity and sporulation class^2,3^ |
|  |  |  | EU-D-42 | 16.5, 22.8* | 50 |  |  |
|  | CH04h02_Ga | ‘Gala’** × ‘Dülmener Rosenapfel’ | Five isolates* | 18.9 | 54.8 | ^6^ | Isolates EU-B04, EU-NL19,EU-D42, EU-NL24 and 1066 |
|  | CH02d12 | ‘Fiesta’** × ‘Discovery’** | Multiple strains | 11 | 55 | ^7^ |  |
|  |  | TN10-8 × Fiesta** | EU-B04 | 18.1 | 65.1 | ^5^ |  |
|  |  |  | 09BCZ014 (2557) | 7.9 | 47.5 |  |  |
| LG12 | *Rvi1* | ‘Discovery’** × ‘TN10-8’ | 1066 | 82.7 | 84 | ^4^ |  |
|  |  | ‘Fiesta’** × ‘Discovery’ | Multiple strains | 6 | 55 | ^7^ |  |
|  |  | TN10-8 × Fiesta** | EU-B04 | 3.7 | 65.1 | ^5^ |  |
| LG13 | E32AM58-23 | ‘Discovery’** × ‘TN10-8’ | EU-B04 | 5.5 | 61 | ^4^ |  |
|  |  | TN10-8** × Fiesta | EU-B04 | 9.3 | 65.1 | ^5^ |  |
|  |  |  | 09BCZ014 (2557) | 10.1 | 47.5 |  |  |
| LG15 | PB610b | ‘Prima'** × 'Fiesta’ | 302 | 5.6, 6.0* | 70 | ^1^ | Explained variance for severity and sporulation class^2,3^ |
|  | E32AM59-1 | ‘Fiesta’** × ‘Discovery’ | Multiple strains | 7 | 16 | ^7^ |  |
|  |  | ‘Discovery’** × ‘TN10-8’ | EU-NL24 | 7.7 | 38 | ^4^ |  |
| LG17 | CH01h01, MC228c | ‘Prima' × 'Fiesta’** | 302 | 13.4, 11.0* | 70 | ^1^ | Explained variance for severity and sporulation class^2,3^ |
|  |  |  | EU-D-42 | 9.9, 9.4* | 50 |  |  |
|  | CH01h01 | ‘Gala’** × ‘Dülmener Rosenapfel’** | Five isolates* | 14.4; 5.7 | 54.8 | ^6^ | Isolates EU-B04, EU-NL19,EU-D42, EU-NL24 and 1066 |
|  | E32AM48–17 | ‘Fiesta’** × ‘Discovery’** | Multiple strains | 23, 9 | 55, 16* | ^7^ | Explained variance for leaves and fruit |
|  |  | ‘Discovery’** × ‘TN10-8’ | EU-B04 | 6.3 | 61 | ^4^ |  |
|  |  |  | 104 | 19.4 | 31 |  |  |
|  |  |  | 302 | 13.1 | 38 |  |  |
|  |  | ‘Discovery’** × ‘TN10-8’** | EU-D42 | 9.0 | 13 |  |  |
|  |  |  | EU-NL24 | 10.0 | 38 |  |  |
|  |  | ‘Discovery’** × ‘TN10-8’ | MLI | 4.7 | 48 |  |  |
|  | E33CM50-2 |  | Gd-CH | 19.0 | 27 |  |  |
|  |  | TN10-8 × Fiesta** | EU-B04 | 29 | 65.1 | ^5^ |  |
|  |  |  | 09BCZ014 (2557) | 10.7 | 47.5 | ^5^ |  |

1.	Durel, C. E. *et al.* Genetic dissection of partial resistance to race 6 of *Venturia inaequalis* in apple. *Genome* **46**, 224–234 (2003).

2.	Croxall, H. E., Gwynne, D. C. & Jenkins, J. E. E. The rapid assessment of apple scab on fruit. *Plant Pathol.* **1**, 89–92 (1952).

3.	Chevalier, M., Lespinasse, Y. & Renaudin, S. A microscopic study of the different classes of symptoms coded by the *Vf* gene in apple for resistance to scab (*Venturia inaequalis*). *Plant Pathol.* **40**, 249–256 (1991).

4.	Calenge, F. *et al.* Quantitative trait loci (QTL) analysis reveals both broad-spectrum and isolate-specific QTL for scab resistance in an apple progeny challenged with eight isolates of *Venturia inaequalis*. *Phytopathology* **94**, 370–9 (2004).

5.	Bénéjam, J. *et al.* Acibenzolar-S-methyl and resistance quantitative trait loci complement each other to control apple scab and fire blight. *Plant Dis.* **105**, 1702–1710 (2020).

6.	Soufflet-Freslon, V., Gianfranceschi, L., Patocchi, A. & Durel, C.-E. Inheritance studies of apple scab resistance and identification of *Rvi14*, a new major gene that acts together with other broad-spectrum QTL. *Genome* **51**, 657–67 (2008).

7.	Liebhard, R. *et al.* Mapping quantitative field resistance against apple scab in a ‘Fiesta’ x ‘Discovery’ progeny. *Phytopathology* **93**, 493–501 (2003).
