# Supplementary material for: Phenotyping, genetics, and “-omics” approaches to unravel and introgress enhanced resistance against apple scab (Venturia inaequalis) in apple cultivars (Malus × domestica)
Source: Hortic Res. 2024 Jan 10;11(2):uhae002. doi: 10.1093/hr/uhae002 (PMC10873587; doi:10.1093/hr/uhae002)
Supplement: Web_Material_uhae002 [file web_material_uhae002.zip › REVIEWED Supplementary table 3 List of transgenic apples.doc]

Supplementary table 3 List of transgenic apples developed to study resistance to apple scab

| **Gene** | **Gene origin** | **Accession** | **Tissue** | **Vector** | **Disease resistance** | **Ref.** |
| --- | --- | --- | --- | --- | --- | --- |
| Endochitinase, chitobiosidase | *Trichoderma harzianum* | 'Royal Gala' | Leaf | pB1525 | Resistance to *V. inaequalis* | ^1^ |
| Endochitinase, exochitinase | *Trichoderma atroviride* | ‘Ariane’ and ‘Galaxy’ | Leaf | pBIN | Resistance to *V. inaequalis* | ^2^ |
| *HcrVf1*, *HcrVf2* | *Malus floribunda* 821 | 'Gala' | Leaf | pMF1 | Resistance to *V. inaequalis* | ^3^ |
| *HcrVf2* | *Malus floribunda* 821 | 'Gala' and 'Elstar' | Leaf | pCambia2301 | Resistance to *V. inaequalis* | ^4^ |
| *HcrVf2* | *M. x domestica* ‘Florina’ | 'Gala' | Leaf | pMF1 | Resistance to *V. inaequalis* | ^5^ |
| *MpNPR1* | *M. × domestica* | ‘Galaxy’ and ‘M26’ | Leaf | pKYLXAtNPR1, pKYLX35SMpNPR1, or pKYLXPINMpN-PR1 | Resistance to *V. inaequalis* and *Gymnosporangium juniperi-virginianae* | ^6^ |
| Puroindoline-B | *Triticum aestivum* | ‘Ariane’ and ‘Galaxy’ | Leaf | pPuro-b | Resistance to *V. inaequalis* | ^7^ |
| *Vr2-A, -B, -C* | *M. × domestica* GMAL 2473 | ‘Gala’ | Leaf | pMF1 | Resistance to *V. inaequalis* | ^8^ |
| *Vfa1*, *Vfa2*, *Vfa4* | *Malus floribunda* 821 | ‘Galaxy’ and ‘McIntosh’ | Leaf | pCAMBIA2301 | Resistance to *V. inaequalis* | ^9^ |
| Chalcone-3-hydroxylase | *Cosmos sulphureus* | ‘Pinova’ | Axillary shoot | p9u10-35S::CH3H | Resistance to *V. inaequalis* and *Erwinia amylovora* | ^10^ |
| Leaf colour *(Lc)* | *Zea mays* | ‘Holsteiner Cox’ | Nodal segments | pBI121 | Resistance to *V. inaequalis* and *Erwinia amylovora* | ^11^ |

1.	Wong, K.-. W., Harman, G. E., Norelli, J. L., Gustafson, H. L. & Aldwinckle, H. S. Chitinase-transgenic lines of ‘royal gala’ apple showing enhanced resistance to apple scab. *Acta Hortic.* **484**, 595–600 (1999).

2.	Faize, M. *et al.* Chitinases of *Trichoderma atroviride* induce scab resistance and some metabolic changes in two cultivars of apple. *Phytopathology* **93**, 1496–504 (2003).

3.	Joshi, S. G. *et al.* Functional analysis and expression profiling of *HcrVf1* and *HcrVf2* for development of scab resistant cisgenic and intragenic apples. *Plant Mol. Biol.* **75**, 579–591 (2011).

4.	Szankowski, I., Waidmann, S., Silfverberg-dilworth, E., Broggini, G. & Gessler, C. Highly scab-resistant transgenic apple lines achieved by introgression of *HcrVf2* controlled by different native promoter lengths. *Tree Genet. Genomes* **5**, 349–358 (2009).

5.	Vanblaere, T. *et al.* The development of a cisgenic apple plant. *J. Biotechnol.* **154**, 304–311 (2011).

6.	Malnoy, M., Jin, Q., Borejsza-Wysocka, E. E., He, S. Y. & Aldwinckle, H. S. Overexpression of the apple *MpNPR1* gene confers increased disease resistance in *Malus* x *domestica*. *Mol. Plant-Microbe Interact.* **20**, 1568–1580 (2007).

7.	Faize, M. *et al.* Expression of wheat puroindoline-b reduces scab susceptibility in transgenic apple (*Malus × domestica* Borkh.). *Plant Sci.* **167**, 347–354 (2004).

8.	Schouten, H. J. *et al.* Cloning and functional characterization of the *Rvi15* (*Vr2*) gene for apple scab resistance. *Tree Genet. Genomes* **10**, 251–260 (2014).

9.	Malnoy, M., Xu, M., Borejsza-wysocka, E., Korban, S. S. & Aldwinckle, H. S. Two receptor-like genes, *Vfa1* and *Vfa2*, confer resistance to the fungal pathogen *Venturia inaequalis* inciting apple scab disease. *Mol. Plant Microbe Interact.* **21**, 448–458 (2008).

10.	Hutabarat, O. S. *et al.* Transgenic apple plants overexpressing the chalcone 3-hydroxylase gene of *Cosmos sulphureus* show increased levels of 3-hydroxyphloridzin and reduced susceptibility to apple scab and fire blight. *Planta* **243**, 1213–1224 (2016).

11.	Flachowsky, H. *et al.* Transgenic apple plants overexpressing the *Lc* gene of maize show an altered growth habit and increased resistance to apple scab and fire blight. *Planta* **231**, 623–635 (2010).
