# Supplementary material for: Phenotyping, genetics, and “-omics” approaches to unravel and introgress enhanced resistance against apple scab (Venturia inaequalis) in apple cultivars (Malus × domestica)
Source: Hortic Res. 2024 Jan 10;11(2):uhae002. doi: 10.1093/hr/uhae002 (PMC10873587; doi:10.1093/hr/uhae002)
Supplement: Web_Material_uhae002 [file web_material_uhae002.zip › REVIEWED Supplementary table 4 Analysis of differential gene expression.doc]

Supplementary table 4 Overview of reports on the analysis of differential gene expression in apple prior or after *V. inaequalis* inoculation using various methods. DEGs – differentially expressed genes; TDFs – transcript derived fragments; ESTs – expressed sequence tags.

| **Method** | **Accession** | **Tissue** | **Process** | **No. of identified transcripts/differentially expressed genes (DEGs)/transcription-derived fragments (TDFs)/expressed sequence tags (ESTs)** | **Ref.** |
| --- | --- | --- | --- | --- | --- |
| RT-qPCR | ‘Remo' and 'Elstar' | Leaves | Monogenic resistance | 8 transcripts | ^1^ |
| RT-qPCR | ‘Royal Gala’ | Leaves | Susceptibility | 1 transcript | ^2^ |
| RT-qPCR | ‘Golden Delicious’ | Leaves | Ontogenic resistance | 5 DEGs | ^3^ |
| RT-qPCR | ‘Président Roulin’ and ‘Golden Delicious’ | Leaves | Polygenic resistance | 13 candidate defense genes | ^4^ |
| RT-qPCR | ‘Gala’ and *HcrVf2*-‘Gala | Leaves | Monogenic resistance | 7 DEGs | ^5^ |
| Northern blot | ‘Golden Delicious’ and ‘Florina’ | Leaves | Monogenic resistance | 1 transcript | ^6^ |
| cDNA-AFLP | ‘Président Roulin’ and ‘Gala’ | Leaves | Polygenic resistance | 467 TDFs | ^7^ |
| cDNA-AFLP | *HcrVf2* 'Gala' | Leaves | Monogenic resistance | 66 TDFs | ^8^ |
| Microarray | ‘Golden Delicious’ and ‘Rewena’ | Leaves | Monogenic resistance | 17 DEGs | ^9^ |
| Suppression subtractive hybridization | ‘Golden Delicious’ and ‘Florina’ | Leaves | Monogenic resistance | 48 TDFs | ^10^ |
| Suppression subtractive hybridization | ‘Remo’ and ‘Elstar’ | Leaves | Monogenic resistance | 480 ESTs | ^1^ |
| Suppression subtractive hybridization | ‘Gala’ and *HcrVf2*-‘Gala | Leaves | Monogenic resistance | 523 ESTs | ^5^ |
| RNA sequencing | ‘Golden Delicious’ | Leaves | Ontogenic scab resistance | 5823 DEGs | ^3^ |
| RNA sequencing | ‘Gala’ and *HcrVf2*-‘Gala | Leaves | Monogenic resistance | 3,334 DEGs | ^11^ |
| Northern blot | ‘Royal Gala’ | Leaves | Susceptibility | 259 identified sequences | ^2^ |

1.	Degenhardt, J. & Nasser, Æ. A. Characterization by suppression subtractive hybridization of transcripts that are differentially expressed in leaves of apple scab-resistant and susceptible cultivars of *Malus domestica*. *Mol. Genet. Genomic* **273**, 326–335 (2005).

2.	Girardi, C. L. *et al.* Genome-wide analysis of the AP2/ERF superfamily in apple and transcriptional evidence of ERF involvement in scab pathogenesis. *Sci. Hortic. (Amsterdam).* **151**, 112–121 (2013).

3.	Gusberti, M., Gessler, C. & Broggini, G. A. L. RNA-Seq analysis reveals candidate genes for ontogenic resistance in *Malus-Venturia* pathosystem. *PLoS One* **8**, e78457 (2013).

4.	Bastiaanse, H., Muhovski, Y., Mingeot, D. & Lateur, M. Candidate defense genes as predictors of partial resistance in ‘Président Roulin’ against apple scab caused by *Venturia inaequalis*. *Tree Genet. Genomes* **11**, 125 (2015).

5.	Paris, R. *et al.* Expression profiling in *HcrVf2*-transformed apple plants in response to *Venturia inaequalis*. *Tree Genet. Genomes* **5**, 81–91 (2009).

6.	Komjanc, M. *et al.* A leucine-rich repeat receptor-like protein kinase (*LRPKm1*) gene is induced in *Malus* × *domestica* by *Venturia inaequalis* infection and salicylic acid treatment. *Plant Mol. Biol.* **40**, 945–957 (1999).

7.	Bastiaanse, H. *et al.* Gene expression profiling by cDNA-AFLP reveals potential candidate genes for partial resistance of ‘Président Roulin’ against *Venturia inaequalis*. *BMC Genomics* **15**, 1043 (2014).

8.	Paris, R. *et al.* dHPLC efficiency for semi-automated cDNA-AFLP analyses and fragment collection in the apple scab-resistance gene model. *Planta* **235**, 1065–1080 (2012).

9.	Holzapfel, C., Meisel, B., Thummler, F., Leser, C. & Treutter, D. Differential gene expression in leaves of a scab susceptible and a resistant apple cultivar upon *Venturia inaequalis* inoculation. *Trees - Struct. Funct.* **26**, 121–129 (2012).

10.	Cova, V. *et al.* Apple genes involved in the response to *Venturia inaequalis* and salicylic acid treatment. *Sci. Hortic. (Amsterdam).* **226**, 157–172 (2017).

11.	Perchepied, L. *et al.* Successful intergeneric transfer of a major apple scab resistance gene (*Rvi6*) from apple to pear and precise comparison of the downstream molecular mechanisms of this resistance in both species. *BMC Genomics* **22**, 1–18 (2021).
