# Supplementary material for: Phenotyping, genetics, and “-omics” approaches to unravel and introgress enhanced resistance against apple scab (Venturia inaequalis) in apple cultivars (Malus × domestica)
Source: Hortic Res. 2024 Jan 10;11(2):uhae002. doi: 10.1093/hr/uhae002 (PMC10873587; doi:10.1093/hr/uhae002)
Supplement: Web_Material_uhae002 [file web_material_uhae002.zip › REVIEWED Supplementary table 5 Transcriptomics processes.doc]

Supplementary table 5 Summary of transcriptomic analyses in susceptible and resistant apple plants in relation to scab inoculation, i.e. before and upon inoculation, indicating specific affected genes and mechanisms.

| **Comparison** | **Time points** | **Regulation** | **Molecular process and corresponding genes** | **Cultivars** |
| --- | --- | --- | --- | --- |
| **Resistant vs. susceptible** | Before inoculation | **↑**Upregulation | **Defense proteins** (e.g. endochitinase class III PR3^1,2^, β-1,3 glucanase PR2, wound-responsive protein-related, major allergen Mal d 1^2^) | ‘Remo’ vs. ‘Elstar’^2^ |
|  |  |  | **ROS accumulation** (e.g. metallothioneins, manganese superoxide dismutase^2^) |  |
|  |  |  | **Photosynthesis-related proteins** (e.g. Cytochrome b6) |  |
|  |  | **↓**Downregulation | **Defense proteins** (e.g. Bet v I allergen^2^) | ‘Remo’ vs. ‘Elstar’^2^ |
|  |  |  | **ROS accumulation** (e.g. catalase, manganese superoxide dismutase^2^) |  |
|  |  |  | **Photosynthesis-related proteins** (e.g. ribulose 1,5-bisphosphate carboxylase, ferredoxin, NADH2 dehydrogenase (ubiquinone), Fructose-bisphosphate aldolase^2^) |  |
|  |  |  | **Primary and specializedmetabolism** (e.g. GDSL lipase/esterase^1,2^, lipid-transfer protein (Mal d3)^1^) |  |
|  | Upon inoculation | **↑**Upregulation | **Pathogen reception** (e.g. LRR receptor-like protein kinase LRPKm1^3^, *HcrVf2*^1^, CERK1, proline extensin-like receptor kinase 1(PERK1)^4^) | ‘Florina’ vs. ‘Golden Delicious’^3^, *HcrVf2* 'Gala' vs. 'Gala'^1,4^ |
|  |  |  | **Signaling** (e.g. EIN3-like protein 2 (EIL2)^1^, G-proteins, calmodulin dependent protein kinase (CPK), calcium ion binding, auxin signaling, jasmonic acid signaling, brassinosteroid signaling, salycilyc acid signaling, systemic acquired resistance signaling^4^) | *HcrVf2* 'Gala' vs. 'Gala'^1,4^ |
|  |  |  | **Transcription** (e.g. elongation factor-1α, actin^1^) | *HcrVf2* 'Gala' vs. 'Gala'^1^ |
|  |  |  | **Defense proteins** (e.g. EchIII^1^) |  |
|  |  |  | **ROS accumulation and lignification** (e.g. cytosolic ascorbate peroxidase, cinnamyl alcohol dehydrogenase^5^, lignin biosynthesis genes^4^) | ‘Rewena’ vs. ‘Golden Delicious’^5^, *HcrVf2* 'Gala' vs. 'Gala'^4^ |
|  |  |  | **Protein metabolism** (e.g. cysteine protease 1^5^, ubiquitin-ligase E3^4^) | ‘Rewena’ vs. ‘Golden Delicious’^5^, *HcrVf2* 'Gala' vs. 'Gala^4^ |
|  |  |  | **Primary and specialized metabolism** (e.g. UDP-glucuronosyl/UDP glucosyltransferase (UGT)^1^, NAPH-dependent hydroxypyruvate reductase^5^, phenylalanine ammonia-lyase, cinnamate 4-hydroxylase and chalcone synthase, flavonoid and lignin biosynthesis genes^4^) | *HcrVf2* 'Gala' vs. 'Gala'^1,4^, ‘Rewena’ vs. ‘Golden Delicious’^5^ |
|  |  | **↓**Downregulation | **Signaling** (e.g. auxin-repressed protein^5^, jasmonic acid signaling, MYB signaling^4^) | ‘Rewena’ vs. ‘Golden Delicious’^5^, *HcrVf2* 'Gala' vs. 'Gala^4^ |
|  |  |  | **Protein metabolism** (e.g. cysteine proteinase^5^) | ‘Rewena’ vs. ‘Golden Delicious’^5^ |
|  |  |  | **Primary and specialized metabolism** (e.g. GDSL lipase/esterase^1^, spermidine synthase, P450 hydroxylase^5^, MYB repressor, flavonoid and lignin biosynthesis genes^4^) | *HcrVf2* 'Gala' vs. 'Gala'^1^, ‘Rewena’ vs. ‘Golden Delicious’^5^ |
| ***V. inaequalis*- vs. uninoculated susceptible genotypes** | Upon inoculation | **↑**Upregulation | **Pathogen recognition** (e.g. LRR receptor-like protein kinase LRPKm1^3^, CC-NBS-LRR and LRR receptor kinase-like protein^6,7^) | ‘Golden Delicious’^3^, 'Gala'^6,7^ |
|  |  |  | **Signaling** (e.g. genes encoding MAP kinase and zinc finger homeodomain protein 1^6,7^) | 'Gala'^6,7^ |
|  |  |  | **Defense proteins** (e.g. thaumatin-like protein, P450 hydroxylase^5^, chitinase a type II (*MdchiII*), chitinase type V (*MdchiV*)^8^, cystein protease inhibitor, cystein protease, chitinases, lysosomal Pro-X carboxypeptidase, pectin methylesterase inhibitor^6,7^) | ‘Golden Delicious’^5,8^, ‘Gala’^6,7^ |
|  |  |  | **Redox and/or lignin biosynthesis** (e.g. tocopherol cyclase, peroxidase 12^6,7^) | 'Gala'^6,7^ |
|  |  |  | **Primary and specialized metabolism** (e.g. glyceraldehyde-3-phosphate dehydrogenase^5^, cysteine protease inhibitor, cysteine protease, chitinase^6,7^, phenylpropanoid biosynthesis genes, chalcone synthase^9^) | ‘Gala’^6,7^, ‘Golden Delicious’^5,9^ |
|  |  | **↓**Downregulation | **Defense proteins** (e.g. cystein protease, pectin methylesterase inhibitor^6,7^, xyloglucan endotransglycosylase^8^) | ‘Gala’^6,7^, ‘Golden Delicious’^8^ |
|  |  |  | **Redox and/or lignin biosynthesis** (e.g. peroxidase 12^6,7^, peroxidase (*MdPrx*)^8^) | 'Gala'^6,7^, ‘Golden Delicious’^8^ |
|  |  |  | **Primary and specialized metabolism** (e.g. glutamine synthase, cinnamate 4-hydroxylase^8^, major allergen and lipid transfer protein Mal d 3^5^) | ‘Golden Delicious’^5,8^ |
| ***V. inaequalis*- vs. uninoculated resistant genotypes** | Upon inoculation | **↑**Upregulation | **Pathogen recognition** (e.g. LRR receptor-like protein kinase LRPKm1^3^, MbR7, ARGA4, leucine-rich repeat (LRR) receptor-like protein kinase 2 (LRPK2), LRPK^10^, CC-NBS-LRR and LRR receptor kinase-like protein^6,7^) | ‘Florina’^3^, *HcrVf2*-'Gala'^10^, ‘Président Roulin’^6,7^ |
|  |  |  | **Signaling** (e.g. genes encoding MAP kinase and zinc finger homeodomain protein 1^6,7^, transducin/WD40-repeat containing protein^10^, and *MD14-3-3*^8^) | ‘Président Roulin’, *HcrVf2*-'Gala'^10^, 'Florina'^8^ |
|  |  |  | **Pathogen defense** (e.g., CAD1^10^, chitinase a type II (*MdchiII*), xyloglucan endotransglycosylase^8^, pectin methylesterase inhibitor^6,7^, cystein protease inhibitor, chitinases^6,7^) | *HcrVf2*-'Gala'^10^, Président Roulin’^6,7^, ‘Florina’^8^ |
|  |  |  | **Redox and/or lignin biosynthesis** (e.g. cytochrome P450, tocopherol cyclase^6,7^, peroxidase 12^6,7^) | ‘Président Roulin’^6,7^ |
|  |  |  | **Primary and specialized metabolism** (e.g. cysteine protease inhibitor^6,7^, acetohydroxy acid isomeroreductase (AAIR)^10^) | ‘Président Roulin’^6,7^, *HcrVf2*-'Gala'^10^ |
|  |  | **↓**Downregulation | **Pathogen defense** (e.g.lysosomal Pro-X carboxypeptidase, cystein protease, cystein protease inhibitor^6,7^, chitinase type V (*MdchiV*)^8^) | ‘Président Roulin’^6,7^, ‘Florina’^8^ |
|  |  |  | **Redox and/or lignin biosynthesis** (e.g. metallothionein type 2-like (*MTH2L*)^2^, cytochrome P450^6,7^, peroxidase (*MdPrx*)^8^) | *HcrVf2*-'Gala'^10^, ‘Président Roulin’^6,7^, ‘Florina’^8^ |
|  |  |  | **Primary and specialized metabolism** (e.g. glycosyl hydrolase 1, β-glucosidase 44 (*β-Glu44*), ethylene-dependent gravitropism-deficient and yellow-green 1, 3-deoxy-D-arabinoheptulosonate 7-phosphate synthase 2 (DAHPS2), ATP synthase ATPG^10^) | *HcrVf2*-'Gala'^10^ |
| **Ontogenic-resistant vs. susceptible** | Upon inoculation | **↑**Upregulation | **Redox genes and defense-related production of metabolites** (e.g. peroxidase, lipid transfer proteins, lipoxygenase, and cytochrome P450^9^) | ‘Golden Delicious’^9^ |
|  |  |  | **Biosynthesis of cell wall, callose, wax, and lignin precursors** (e.g. callose synthase) |  |
|  |  | **↓**Downregulation | **Defense proteins** (e.g. Enhanced Disease Susceptibility 1) | ‘Golden Delicious’^9^ |

1.	Paris, R. *et al.* Expression profiling in *HcrVf2*-transformed apple plants in response to *Venturia inaequalis*. *Tree Genet. Genomes* **5**, 81–91 (2009).

2.	Degenhardt, J. & Nasser, Æ. A. Characterization by suppression subtractive hybridization of transcripts that are differentially expressed in leaves of apple scab-resistant and susceptible cultivars of *Malus domestica*. *Mol. Genet. Genomic* **273**, 326–335 (2005).

3.	Komjanc, M. *et al.* A leucine-rich repeat receptor-like protein kinase (*LRPKm1*) gene is induced in *Malus* × *domestica* by *Venturia inaequalis* infection and salicylic acid treatment. *Plant Mol. Biol.* **40**, 945–957 (1999).

4.	Perchepied, L. *et al.* Successful intergeneric transfer of a major apple scab resistance gene (*Rvi6*) from apple to pear and precise comparison of the downstream molecular mechanisms of this resistance in both species. *BMC Genomics* **22**, 1–18 (2021).

5.	Holzapfel, C., Meisel, B., Thummler, F., Leser, C. & Treutter, D. Differential gene expression in leaves of a scab susceptible and a resistant apple cultivar upon *Venturia inaequalis* inoculation. *Trees - Struct. Funct.* **26**, 121–129 (2012).

6.	Bastiaanse, H., Muhovski, Y., Mingeot, D. & Lateur, M. Candidate defense genes as predictors of partial resistance in ‘Président Roulin’ against apple scab caused by *Venturia inaequalis*. *Tree Genet. Genomes* **11**, 125 (2015).

7.	Bastiaanse, H. *et al.* Gene expression profiling by cDNA-AFLP reveals potential candidate genes for partial resistance of ‘Président Roulin’ against *Venturia inaequalis*. *BMC Genomics* **15**, 1043 (2014).

8.	Cova, V. *et al.* Apple genes involved in the response to *Venturia inaequalis* and salicylic acid treatment. *Sci. Hortic. (Amsterdam).* **226**, 157–172 (2017).

9.	Gusberti, M., Gessler, C. & Broggini, G. A. L. RNA-Seq analysis reveals candidate genes for ontogenic resistance in *Malus-Venturia* pathosystem. *PLoS One* **8**, e78457 (2013).

10.	Paris, R. *et al.* dHPLC efficiency for semi-automated cDNA-AFLP analyses and fragment collection in the apple scab-resistance gene model. *Planta* **235**, 1065–1080 (2012).
