# Supplementary material for: Phenotyping, genetics, and “-omics” approaches to unravel and introgress enhanced resistance against apple scab (Venturia inaequalis) in apple cultivars (Malus × domestica)
Source: Hortic Res. 2024 Jan 10;11(2):uhae002. doi: 10.1093/hr/uhae002 (PMC10873587; doi:10.1093/hr/uhae002)
Supplement: Web_Material_uhae002 [file web_material_uhae002.zip › REVIEWED Supplementary table 6 Summary of omics approaches.doc]

Table 6 Summary of omics approaches used in commercial apple cultivars. Proteins and metabolites that were identified under different comparisons are indicated in bold

| **Cultivar** | **Phenotype/ Genotype** | **Transcriptomics** | **Proteomics** | **Metabolomics** |
| --- | --- | --- | --- | --- |
| ‘Golden Delicious’ | Susceptible  *Rvi1* gene (considered non-functional) | Inoculation:  Upregulated: LRR-RLP kinase^1^, **thaumatin-like protein**, **P450 hydroxylase**, glyceraldehyde-3-phosphate dehydrogenase, heat shock protein 82^2^, **cysteine proteinase inhibitor**, **chalcone synthase**^3^, putative chitinase a type II (*MdchiII*), putative xyloglucan endotransglycosylase (*MdXET*), and **chitinase** type V (*MdchiV*)^4^  Downregulated: **Mal d 3**^2^, **cysteine protease**^3^, putative **peroxidase** (*MdPrx*), the cinnamate 4-hydroxylase (*MdC4H*), and *MdXET*^4^  Comparison with a resistant cultivar:  Upregulated: annexin, auxin-repressed protein like-protein, small Ras-like GTP-binding protein, **cysteine proteinase**, **P450 hydroxylase**, **spermidine synthase**^2^  Downregulated: LRR-RLP kinase^1^, oxygen evolving enhancer protein 2, NAPH-dependent hydroxypyruvate reductase, polypeptide of the oxygen-evolving complex of photosystem II, cytosolic ascorbate **peroxidase**, putative cinnamyl alcohol dehydrogenase, photosystem I reaction centre subunit X psaK, **cysteine protease 1**^2^  Ontogenicity:  Upregulated in old leaves: **metallothionein-3-like protein**, **lipoxygenase**, **lipid transfer protein**, and **peroxidase** 3^3^  Downregulated in old leaves: **enhanced disease susceptibility 1**^3^ | Comparison with a resistant cultivar:  Upregulated: monodehydroascorbate reductase, light-harvesting complex I protein Lhca1, light-harvesting complex II protein Lhcb1, 3-dehydroquinate synthase, glycyl-tRNA synthetase, protein THYLAKOID FORMATION1, ATP-citrate synthase …^5^  Downregulated: **Mal d 1-like**, **major allergen Mal d 1 (PR-10)**, apple phosphoglyceromutase, esterase/lipase domain-containing protein, protein disulfide isomerase, phosphoglycerate kinase, linoleate 13s-**lipoxygenase** 2- chloroplastic, **chalcone synthase** …^5^ | Inoculation:  Upregulated: TPC, **flavonols**, chlorogenic acid, chlorogenic acid, **p-coumaric acid**, caffeic acid, **catechin**, epicatechin, rutin, quercetin-3-rhamnoside^6^, ferulic acid, phloretin^7^, **hydroxycinnamic acids**, **procyanidins**^8^, dihydrochalcones, **flavanols**^9^, phloridzin^7,10^  Downregulated: procyanidins, quercetins and phloretin^10^  Comparison with a resistant cultivar:  Upregulated: **hydroxycinnamic acids**, **phloridzin**^8^  Downregulated: **p-coumaric acid**, **flavanols**^11^, **flavonols**^9^, **catechin**, **procyanidins**^8^, phenol 173^12^ |
| ‘Elstar’ | Susceptible (No *Rvi* gene) | Inoculation:  **Metallothionein type 3**^13^  Comparison with a resistant cultivar:  Upregulated: GDSL-motif lipase/hydroxylase,**chalcone synthase** 11 and a Bet v I allergen type protein, small subunit of ribulose-1,5-bisphosphat carboxylase/oxygenase and acid phosphatase^13^  Downregulated: **β-1,3-glucanase**, **cysteine protease inhibitor**, **endochitinase**, wound-responsive protein, ferrochelatase, **ribonuclease-like PR-10b**, and ADP-ribosylation factor^13^ | Inoculation:  Upregulated: **β-1,3-glucanase**, **chitinase**, **thaumatin-like protein**, **cysteine-like protease**^14^  Comparison with a resistant cultivar:  Downregulated: **β-1,3-glucanase**, **chitinase**, **thaumatin-like protein**, **cysteine-like protease**^14^ |  |
| ‘(Royal) Gala’ | Susceptible  No *Rvi* gene  QRLs (CH01h01, CH04h02_Ga) | Inoculation:  Upregulated: ERF MDP0000128979^15^, cc-nbs-lrr resistance protein, LRR receptor kinase-like protein, 2-cys **peroxiredoxin**, **MAP kinase,** ABC transporter, **cytochrome P450**, **peroxidase** 12, tocopherol cyclase, **cysteine protease inhibitor**, **chitinase**, zinc finger homeodomain protein1, pectin methylesterase inhibitor^16^, cc-nbs-lrr resistance protein, LRR receptor kinase-like protein, **MAP kinase**, zinc finger homeodomain protein1, **chitinase**, pectin methylesterase inhibitor, **cysteine protease**, **cysteine protease inhibitor**, tocopherol cyclase^17^  Downregulated: *HcrVf* paralog, phi class glutathione, **cysteine protease transferase**^16^  A comparison with a resistant cultivar:  Upregulated: TMV resistance protein, ABC transporter, cytochrome P450, lysosomal Pro-X carboxypeptidase^16^, **cysteine protease**, lysosomal Pro-X carboxypeptidase^17^, non-specific lipid-transfer protein precursor (Mal d 3), and putative GDSL-motif lipase/acylhydrolase (GDSL)^18^  Downregulated: cc-nbs-lrr resistance protein, *HcrVf* paralog, LRR receptor kinase-like protein, 2-cys **peroxiredoxin**, **MAP kinase**, **cytochrome P450**, uroporphyrinogen decarboxylase, **peroxidase** 12, tocopherol cyclase, **cysteine protease inhibitor**, **chitinase**, zinc finger homeodomain protein 1, pectin methylesterase inhibitor^16^, UDP-glucuronosyl/UDPglucosyltransferase (UGT), class III endo-**chitinase** (EchIII), EIN3-like protein 2 (EIL2), and HcrVf2^4^ |  |  |
| ‘Topaz’ | Resistant (*Rvi6* gene) |  | Inoculation:  Upregulated: **major allergen Mal d 1** (PR-10), **chalcone synthase, enhanced disease susceptibility 1**, heat shock protein 70, linoleate 13s-**lipoxygenase** 2- chloroplastic, heat shock protein 60, TCP (T-complex protein) domain class transcription factor, thioredoxin h, nucleoside diphosphate kinase 1 (NDPK I), bifunctional purine biosynthesis protein, phosphofructokinase,  **beta-glucosidase** 24-like, pyruvate kinase,  pyruvate decarboxylase ...^5^  Downregulated: fructose-1,6-bisphosphatase, glyceraldehyde-3-phosphate dehydrogenase, phosphoribulokinase, fructose-bisphosphate aldolase, light-harvesting complex II protein Lhcb1, oxygen-evolving enhancer protein 1, glycine dehydrogenase ...^5^  A comparison with a susceptible cultivar:  Upregulated: **Mal d 1-like**, **major allergen Mal d 1 (PR-10)**, apple phosphoglyceromutase, esterase/lipase domain-containing protein, protein disulfide isomerase, phosphoglycerate kinase, linoleate 13s-**lipoxygenase** 2- chloroplastic, **chalcone synthase** …^5^  Downregulated: monodehydroascorbate reductase, light-harvesting complex I protein Lhca1, light-harvesting complex II protein Lhcb1, 3-dehydroquinate synthase, glycyl-tRNA synthetase, protein THYLAKOID FORMATION1, ATP-citrate synthase …^5^ | A comparison with a susceptible cultivar:  Upregulated: **p-coumaric acid**, epicatechin, **catechins**, protocatechuic acid, quercetin-3-rhamnoside, phenol 173^12,19^ |
| ‘Rewena’ | Resistant (*Rvi6* gene) | Inoculation:  Upregulated: (**spermidine synthase**)^2^  A comparison with a susceptible cultivar:  Upregulated: oxygen evolving enhancer protein 2, NAPH-dependent hydroxypyruvate reductase, polypeptide of the oxygen-evolving complex of photosystem II, cytosolic ascorbate peroxidase, putative cinnamyl alcohol dehydrogenase, photosystem I reaction centre subunit X psaK, **cysteine protease 1**^2^  Downregulated: annexin, auxin-repressed protein like-protein, small Ras-like GTP-binding protein, cysteine proteinase, **P450 hydroxylase**, spermidine synthase^2^ |  | Inoculation:  Upregulated: **hydroxycinnamic acids**^8^,  A comparison with a susceptible cultivar:  Upregulated: **catechins**, **procyanidins**^8^  Downregulated: **hydroxycinnamic acids**, **phloridzin**^8^ |
| ‘Remo' | Resistant (*Rvi6* gene) | A comparison with a susceptible cultivar:  Upregulated: **β-1,3-glucanase**, **cysteine protease inhibitor**, **endochitinase**, wound-responsive protein, ferrochelatase, **ribonuclease-like PR-10b**, and ADP-ribosylation factor^13^  Downregulated: GDSL-motif lipase/hydroxylase,**chalcone synthase** 11 and a Bet v I allergen type protein. small subunit of ribulose-1,5-bisphosphat carboxylase/oxygenase and acid phosphatase^13^ | Inoculation:  Upregulated: **β-1,3-glucanase**, **chitinase**, **thaumatin-like protein**, **cysteine-like protease**^14^  A comparison with a susceptible cultivar:  Upregulated: **β-1,3-glucanase**, **chitinase**, **thaumatin-like protein**, **cysteine-like protease**^14^ |  |

^*^ List of studied apple varieties with more limited description of defense response to apple scab in different omics: ‘Holsteiner Cox’, ‘Fuji’, ‘Florina’, ‘Prima’, ‘Perico’, ‘Riega’, ‘Pinova’ ‘Remura’, Crabapples, ‘Goldrush’, ‘Goldstar’ ‘Rubinola’, ‘Priscilla’,‘Hansen’s baccata #2’, ‘Dülmener, Rosenapfel’, Antonovka APF22, ‘Geneva’, B45, ‘Durello di Forli’, ‘Honeycrisp’, *Malus* × *floribunda* 821, ‘Ariane’, ‘Galaxy’, ‘Gala’, ‘Zisai Pearl’, ‘Discovery’, *Malus baccata* jackii, Russian apple TSR34T15, *Malus micromalus,* Russian seedling TSR33T239, *Malus pumila* R12740-7A (russian seedling), *Malus* × *robusta,* 1980-015-025, GMAL 2473, 9-AR2T196, A722–7, ‘Geneva’, ‘Jonagold’, ‘Perezosa’, ‘Teorica’, ‘Ricu’, ‘Baturra’, ‘Auksis’, ‘Ligol’, ‘Aldas’, ‘Lodel’, ‘Braeburn’, ‘Red Elstar’, *Malus sylvestrys* L., ‘Majda’, ‘Pink Lady’, ‘Granny Smith’

1.	Komjanc, M. *et al.* A leucine-rich repeat receptor-like protein kinase (*LRPKm1*) gene is induced in *Malus* × *domestica* by *Venturia inaequalis* infection and salicylic acid treatment. *Plant Mol. Biol.* **40**, 945–957 (1999).

2.	Holzapfel, C., Meisel, B., Thummler, F., Leser, C. & Treutter, D. Differential gene expression in leaves of a scab susceptible and a resistant apple cultivar upon *Venturia inaequalis* inoculation. *Trees - Struct. Funct.* **26**, 121–129 (2012).

3.	Gusberti, M., Gessler, C. & Broggini, G. A. L. RNA-Seq analysis reveals candidate genes for ontogenic resistance in *Malus-Venturia* pathosystem. *PLoS One* **8**, e78457 (2013).

4.	Cova, V. *et al.* Apple genes involved in the response to *Venturia inaequalis* and salicylic acid treatment. *Sci. Hortic. (Amsterdam).* **226**, 157–172 (2017).

5.	Daniëls, B. Response of apple (*Malus* x *domestica*) to *Venturia inaequalis*, the causal agent of apple scab: A real time PCR and proteomics study. (Katholieke Universiteit Leuven, 2013).

6.	Mikulič-Petkovšek, M., Štampar, F. & Veberič, R. Increased phenolic content in apple leaves infected with the apple scab pathogen. *J. Plant Pathol.* **90**, 49–55 (2008).

7.	Mikulic Petkovsek, M. *et al.* Accumulation of phenolic compounds in apple in response to infection by the scab pathogen, *Venturia inaequalis*. *Physiol. Mol. Plant Pathol.* **74**, 60–67 (2009).

8.	Leser, C. & Treutter, D. Effects of nitrogen supply on growth, contents of phenolic compounds and pathogen (scab) resistance of apple trees. *Physiol. Plant.* **123**, 49–56 (2005).

9.	Slatnar, A. *et al.* Polyphenol metabolism of developing apple skin of a scab resistant and a susceptible apple cultivar. *Trees - Struct. Funct.* **26**, 109–119 (2012).

10.	Mikulič Petkovšek, M., Slatnar, A., Štampar, F. & Veberič, R. Phenolic compounds in apple leaves after infection with apple scab. *Biol. Plant.* **55**, 725–730 (2011).

11.	Picinelli, A., Dapena, E. & Mangas, J. J. Polyphenolic pattern in apple tree leaves in relation to scab resistance. A preliminary study. *J. Agric. Food Chem.* **43**, 2273–2278 (1995).

12.	Usenik, V., Mikulic;-Petkovsek, M., Solar, A. & Stampar, F. Flavonols of leaves in relation to apple scab resistance. *J. Plant Dis. Prot.* **111**, 137–144 (2004).

13.	Degenhardt, J. & Nasser, Æ. A. Characterization by suppression subtractive hybridization of transcripts that are differentially expressed in leaves of apple scab-resistant and susceptible cultivars of *Malus domestica*. *Mol. Genet. Genomic* **273**, 326–335 (2005).

14.	Gau, A., Koutb, M., Piotrowski, M. & Kloppstech, K. Accumulation of pathogenesis-related proteins in the apoplast of a susceptible cultivar of apple (*Malus domestica* cv. Elstar) after infection by *Venturia inaequalis* and constitutive expression of PR Genes in the resistant cultivar Remo. *Eur. J. Plant Pathol.* **110**, 703–711 (2004).

15.	Girardi, C. L. *et al.* Genome-wide analysis of the AP2/ERF superfamily in apple and transcriptional evidence of ERF involvement in scab pathogenesis. *Sci. Hortic. (Amsterdam).* **151**, 112–121 (2013).

16.	Bastiaanse, H. *et al.* Gene expression profiling by cDNA-AFLP reveals potential candidate genes for partial resistance of ‘Président Roulin’ against *Venturia inaequalis*. *BMC Genomics* **15**, 1043 (2014).

17.	Bastiaanse, H., Muhovski, Y., Mingeot, D. & Lateur, M. Candidate defense genes as predictors of partial resistance in ‘Président Roulin’ against apple scab caused by *Venturia inaequalis*. *Tree Genet. Genomes* **11**, 125 (2015).

18.	Paris, R. *et al.* Expression profiling in *HcrVf2*-transformed apple plants in response to *Venturia inaequalis*. *Tree Genet. Genomes* **5**, 81–91 (2009).

19.	Mikulic Petkovsek, M., Stampar, F. & Veberic, R. Parameters of inner quality of the apple scab resistant and susceptible apple cultivars (*Malus domestica* Borkh.). *Sci. Hortic. (Amsterdam).* **114**, 37–44 (2007).
