# Supplementary material for: Phenotyping, genetics, and “-omics” approaches to unravel and introgress enhanced resistance against apple scab (Venturia inaequalis) in apple cultivars (Malus × domestica)
Source: Hortic Res. 2024 Jan 10;11(2):uhae002. doi: 10.1093/hr/uhae002 (PMC10873587; doi:10.1093/hr/uhae002)
Supplement: Web_Material_uhae002 [file web_material_uhae002.zip › REVIEWED Supplementary table 7 Analysis of proteins.doc]

Supplementary table 7 Summary of studies performing analyses of proteins in apple in relation to apple scab using various methods

| **Method** | **Accession** | **Tissue** | **Process** | **Ref.** |
| --- | --- | --- | --- | --- |
| SDS-PAGE, two-dimensional gel electrophoresis and ESI Q-TOF | ‘Remo’ and ‘Elstar’ | Leaves (intercellular washing fluid) | Resistances to apple scab | ^1^ |
| NMR | ‘Royal Gala’ open pollinated seedlings | Leaves | Protein structure of Cin1 protein | ^2^ |
| SDS PAGE and ESI Q-ToF | ‘Holsteiner Cox’ | Leaves (intercellular washing fluid) | Influence of *P. fluorescens* Bk3 on the resistance to apple scab | ^3^ |
| Two-dimensional gel electrophoresis and MALDI-TOF-TOF | ‘Topaz’, ‘Discovery’, ‘Golden Delicious’ | Leaves | Resistance to apple scab | ^4^ |

1.	Gau, A., Koutb, M., Piotrowski, M. & Kloppstech, K. Accumulation of pathogenesis-related proteins in the apoplast of a susceptible cultivar of apple (*Malus domestica* cv. Elstar) after infection by *Venturia inaequalis* and constitutive expression of PR Genes in the resistant cultivar Remo. *Eur. J. Plant Pathol.* **110**, 703–711 (2004).

2.	Mesarich, C. H. *et al.* Structure, dynamics and domain organization of the repeat protein Cin1 from the apple scab fungus. *Biochim. Biophys. Acta - Proteins Proteomics* **1824**, 1118–1128 (2012).

3.	Schmoock, S., Kürkcüoglu, S. & Gau, A. E. Biological control of apple scab and fire blight by the application of the non-pathogenic bacterium Pseudomonas fluorescens Bk3 to the leaf surface. in *Ecofruit-13th International Conference on Cultivation Technique and Phytopathological Problems in Organic Fruit-Growing* 306–309 (2008).

4.	Daniëls, B. Response of apple (*Malus* x *domestica*) to *Venturia inaequalis*, the causal agent of apple scab: A real time PCR and proteomics study. (Katholieke Universiteit Leuven, 2013).
