# Supplementary material for: Phenotyping, genetics, and “-omics” approaches to unravel and introgress enhanced resistance against apple scab (Venturia inaequalis) in apple cultivars (Malus × domestica)
Source: Hortic Res. 2024 Jan 10;11(2):uhae002. doi: 10.1093/hr/uhae002 (PMC10873587; doi:10.1093/hr/uhae002)
Supplement: Web_Material_uhae002 [file web_material_uhae002.zip › REVIEWED Supplementary table 8 List of metabolites.doc]

Supplementary table 8 Summary of metabolites analyzed in different studies with different methods in various apple cultivars in relation to *Venturia inaequalis* infection.

| **Metabolite** | **Apple cultivar** | **Plant tissue** | **Accumulation response** | **Detection method** | **Ref.** |
| --- | --- | --- | --- | --- | --- |
| Aucuparin | 'Florina' and 'Vista Bella' | Stem, suspension cultures | Higher after inoculation of the resistant cultivar | GC-MS | ^1,2^ |
| Caffeic acid | 'Jonagold' and 'Golden delicious' | Leaf | Higher in infected leaves in comparison to healthy leaves | HPLC | ^3^ |
| Caffeic acid | 'Jonagold' and 'Golden delicious' | Pulp and peel | Higher in the infected spot in comparison to healthy parts | HPLC | ^4^ |
| Catechin | 'Jonagold' and 'Golden delicious' | Pulp and peel | Higher in the infected spot in comparison to healthy parts | HPLC | ^4^ |
| Catechin | 'Jonagold' and 'Golden delicious' | Leaf | Higher in infected leaves in comparison to healthy leaves | HPLC | ^3^ |
| Catechin | Five scab-resistant four scab-susceptible varieties | Pulp and peel | Higher in resistant cultivars in comparison to susceptible cultivars | HPLC | ^5^ |
| Catechin | ‘Golden Delicious’ and ‘Rewena’ | Leaf | Higher in the resistant cultivar in comparison to the susceptible cultivar | HPLC | ^6^ |
| Chlorogenic acid | 'Auksis', 'Ligol', 'Aldas', 'Lodel' | Leaf | Lower in resistant cultivars in comparison to susceptible cultivars | HPLC | ^7^ |
| Chlorogenic acid | 'Jonagold' and 'Golden delicious' | Leaf | Higher in infected leaves in comparison to healthy leaves | HPLC | ^3^ |
| Chlorogenic acid | Five scab-resistant four scab-susceptible varieties | Pulp and peel | Higher in resistant cultivars in comparison to susceptible cultivars | HPLC | ^5^ |
| Chlorogenic acid | 'Jonagold' and 'Golden delicious' | Pulp and peel | Higher in the infected spot in comparison to healthy parts | HPLC | ^4^ |
| Epicatechin | 'Jonagold' and 'Golden delicious' | Pulp and peel | Higher in the infected spot in comparison to healthy parts | HPLC | ^4^ |
| Epicatechin | 'Jonagold' and 'Golden delicious' | Leaf | Higher in the infected leaves in comparison to healthy leaves | HPLC | ^3^ |
| Epicatechin | Five scab-resistant four scab-susceptible varieties | Pulp and peel | Higher in resistant cultivars in comparison to susceptible cultivars | HPLC | ^5^ |
| Eriobofuran | 'Florina' and 'Vista Bella' | Stem, suspension cultures | Higher after inoculation of the resistant cultivar | LC-GC | ^1,2^ |
| Ferulic acid | 'Jonagold' and 'Golden delicious' | Pulp and peel | Higher in the infected spot in comparison to healthy parts | HPLC | ^4^ |
| Flavanols | 13 varieties | Leaves and fruit skins | Higher in resistant cultivars in comparison to susceptible cultivars | HPLC and chemical reaction detection | ^8^ |
| Flavanols | Nine varieties | Leaf | Higher in resistant cultivars in comparison to susceptible cultivars | HPLC | ^9^ |
| Flavanols | 'Golden Delicious' | Leaf | Higher in infected leaves in comparison to healthy leaves | HPLC | ^10^ |
| Flavanols | 'Florina' and 'Golden Delicious' | Peel | Higher in the resistant cultivar in comparison to the susceptible cultivar | HPLC | ^11^ |
| Flavonols | 'Florina' and 'Golden Delicious' | Peel | Higher in the resistant cultivar in comparison to the susceptible cultivar | HPLC | ^11^ |
| Gallic acid | Five scab-resistant four scab-susceptible varieties | Pulp and peel | Higher in resistant cultivars in comparison to susceptible cultivars | HPLC | ^5^ |
| Hydroxycinnamic acids | 'Golden Delicious' and 'Rewena' | Leaf | Higher in the susceptible cultivar and in infected leaves in comparison to the resistant cultivar and healthy leaves | HPLC | ^6^ |
| Hydroxycinnamic acids | 'Golden Delicious' | Leaf | Higher in the infected spot in comparison to healthy parts | HPLC | ^10^ |
| Noraucuparin | 'Florina' and 'Vista Bella' | Stem, suspension cultures | Higher after inoculation of the resistant variety | GC-MS | ^1,2^ |
| p-coumaric acid | 'Jonagold' and 'Golden delicious' | Leaf | Higher in infected leaves in comparison to healthy leaves | HPLC | ^3^ |
| p-coumaric acid | Five scab-resistant four scab-susceptible varieties | Pulp and peel | Higher in resistant cultivars in comparison to susceptible cultivars | HPLC | ^5^ |
| p-Coumaric acid | 'Jonagold' and 'Golden delicious' | Pulp and peel | Higher in the infected spot in comparison to healthy parts | HPLC | ^4^ |
| p-coumaric acid | 9 varieties | Leaf | Higher in polygenic resistant cultivars in comparison to susceptible cultivars | HPLC | ^9^ |
| Phenol 173 | 'Topaz', 'Goldstar', 'GoldRush', 'Golden Delicious Weinsberg' and 'Golden Delicious Clone B' | Leaf | Higher in resistant cultivars in comparison to susceptible cultivars | HPLC | ^12^ |
| Phloretin | 'Golden delicious' | Leaf | Higher in healthy leaves in comparison to infected leaves | HPLC | ^10^ |
| Phloridzin | 'Golden delicious' | Leaf | Higher in infected leaves in comparison to healthy leaves | HPLC | ^10^ |
| Phloridzin | 'Golden Delicious' and 'Rewena' | Leaf | Higher in the susceptible cultivar in comparison to the resistant cultivar | HPLC | ^6^ |
| Phloridzin | 'Jonagold' and 'Golden delicious' | Pulp and peel | Higher in the infected spot in comparison to healthy parts | HPLC | ^4^ |
| Phloridzin | 'Jonagold' and 'Golden delicious' | Leaf | Higher in infected leaves in comparison to healthy leaves | HPLC | ^3^ |
| Procyanidins | 'Golden Delicious' and 'Rewena' | Leaf | Higher in the resistant cultivar and in infected leaves in comparison to the susceptible cultivar and healthy leaves | HPLC | ^6^ |
| Procyanidins | 'Golden delicious' | Leaf | Higher in healthy leaves in comparison to infected leaves | HPLC | ^10^ |
| Quercetin-3-rhamnoside | 'Jonagold' and 'Golden delicious' | Pulp and Peel | Higher in the infected spot in comparison to healthy parts | HPLC | ^4^ |
| Quercetin | 'Golden delicious' | Leaf | Higher in infected leaves in comparison to healthy leaves | HPLC | ^10^ |
| Quercitrin | 'Auksis', 'Ligol', 'Aldas', 'Lodel' | Leaf | Higher in resistant cultivars in comparison to susceptible cultivars | HPLC | ^13^ |
| Quercitrin | 'Jonagold' and 'Golden delicious' | Leaf | Higher in infected leaves in comparison to healthy leaves | HPLC | ^3^ |
| Quercitrin | Five scab-resistant four scab-susceptible varieties | Pulp and peel | Higher in resistant cultivars in comparison to susceptible cultivars | HPLC | ^5^ |
| Rutin | Five scab-resistant four scab-susceptible varieties | Pulp and peel | Higher in susceptible cultivars in comparison to resistant cultivars | HPLC | ^5^ |
| Rutin | 'Jonagold' and 'Golden delicious' | Pulp and peel | Higher in the infected spot in comparison to healthy parts | HPLC | ^4^ |
| Rutin | 'Auksis', 'Ligol', 'Aldas', 'Lodel' | Leaf | Lower in resistant cultivars in comparison to susceptible cultivars | HPLC | ^7^ |
| Rutin | 'Jonagold' and 'Golden delicious' | Leaf | Higher in infected leaves in comparison to healthy leaves | HPLC | ^3^ |
| Sugars | Seedlings populations | Leaves | No clear change in sugar contents between more and less susceptible plants treated with fructans | HPLC | ^14^ |
| Total flavonoid content | 'Auksis', 'Ligol', 'Aldas', 'Lodel' | Leaf | Higher in resistant cultivars in comparison to susceptible cultivars | Folin-Ciocalteau  spectrophotometric method | ^7^ |
| Total phenolic compounds | 'Auksis', 'Ligol', 'Aldas', 'Lodel' | Leaf | Higher in resistant cultivars in comparison to susceptible cultivars | Folin-Ciocalteau  spectrophotometric method | ^7^ |
| Total phenolic compounds | 'Jonagold' and 'Golden delicious' | Pulp and peel | Higher in the infected spot in comparison to healthy parts | Folin-Ciocalteau  spectrophotometric method | ^4^ |
| Total phenolic compounds | 'Golden delicious' | Leaf | Higher in infected leaves in comparison to healthy leaves | Folin-Ciocalteau  spectrophotometric method | ^10^ |
| Total phenolic compounds | 'Jonagold' and 'Golden delicious' | Leaf | Higher in infected leaves in comparison to healthy leaves | Folin-Ciocalteau  spectrophotometric method | ^3^ |
| Total phenolic compounds | Five scab-resistant four scab-susceptible varieties | Pulp and peel | Higher in resistant cultivars in comparison to susceptible cultivars | Folin-Ciocalteau  spectrophotometric method | ^5^ |

1.	Sarkate, A. *et al.* Molecular cloning and functional analysis of a biphenyl phytoalexin‑specific O‑methyltransferase from apple cell suspension cultures. *Planta* **249**, 677–691 (2018).

2.	Sarkate, A., Saini, S. S., Teotia, D., Gaid, M. & Mir, J. I. Comparative metabolomics of scab-resistant and susceptible apple cell cultures in response to scab fungus elicitor treatment. *Sci. Rep.* **8**, 1–14 (2018).

3.	Mikulič-Petkovšek, M., Štampar, F. & Veberič, R. Increased phenolic content in apple leaves infected with the apple scab pathogen. *J. Plant Pathol.* **90**, 49–55 (2008).

4.	Mikulic Petkovsek, M. *et al.* Accumulation of phenolic compounds in apple in response to infection by the scab pathogen, *Venturia inaequalis*. *Physiol. Mol. Plant Pathol.* **74**, 60–67 (2009).

5.	Mikulic Petkovsek, M., Stampar, F. & Veberic, R. Parameters of inner quality of the apple scab resistant and susceptible apple cultivars (*Malus domestica* Borkh.). *Sci. Hortic. (Amsterdam).* **114**, 37–44 (2007).

6.	Leser, C. & Treutter, D. Effects of nitrogen supply on growth, contents of phenolic compounds and pathogen (scab) resistance of apple trees. *Physiol. Plant.* **123**, 49–56 (2005).

7.	Liaudanskas, M. *et al.* Phenolic composition and antioxidant activity of *Malus domestica* leaves. *Sci. World J.* **2014**, 1–10 (2014).

8.	Treutter, D. & Feucht, W. The pattern of flavan-3-ols in relation to scab resistance of apple cultivars. *J. Hortic. Sci.* **65**, 511–517 (1990).

9.	Picinelli, A., Dapena, E. & Mangas, J. J. Polyphenolic pattern in apple tree leaves in relation to scab resistance. A preliminary study. *J. Agric. Food Chem.* **43**, 2273–2278 (1995).

10.	Mikulič Petkovšek, M., Slatnar, A., Štampar, F. & Veberič, R. Phenolic compounds in apple leaves after infection with apple scab. *Biol. Plant.* **55**, 725–730 (2011).

11.	Slatnar, A. *et al.* Polyphenol metabolism of developing apple skin of a scab resistant and a susceptible apple cultivar. *Trees - Struct. Funct.* **26**, 109–119 (2012).

12.	Usenik, V., Mikulic;-Petkovsek, M., Solar, A. & Stampar, F. Flavonols of leaves in relation to apple scab resistance. *J. Plant Dis. Prot.* **111**, 137–144 (2004).

13.	Veberic, R. *et al.* Phenolic compounds in some apple (*Malus domestica* Borkh) cultivars of organic and integrated production. *J. Sci. Food Agric.* **85**, 1687–1694 (2005).

14.	Svara, A. *et al.* Sweet Immunity: The Effect of Exogenous Fructans on the Susceptibility of Apple (*Malus* × *domestica* Borkh.) to *Venturia inaequalis*. *Int. J. Mol. Sci.* **21**, 5885 (2020).
