# Supplementary material for: Phenotyping, genetics, and “-omics” approaches to unravel and introgress enhanced resistance against apple scab (Venturia inaequalis) in apple cultivars (Malus × domestica)
Source: Hortic Res. 2024 Jan 10;11(2):uhae002. doi: 10.1093/hr/uhae002 (PMC10873587; doi:10.1093/hr/uhae002)
Supplement: Web_Material_uhae002 [file web_material_uhae002.zip › REVIEWED Supplementary table 9 Molecular markers.doc]

Supplementary table 9 Summary of molecular markers associated with apple scab resistance loci; LG- linkage group; cM - centimorgans

| **Marker name** | **Source (genotype)** | ***Rvi locus*** | **Type** | **LG** | **Distance from gene (cM)** | **Reference** |
| --- | --- | --- | --- | --- | --- | --- |
| B398-480 | ‘Antonovka’ PI 172633 | *Rvi10* | RAPD | 1 | 16.0 | ^1^ |
| CH-Vf1 | ‘Antonovka’ APF22 | *Rvi17* | SSR | 1 | 1 | ^2^ |
| CH-Vf1 | *Malus floribunda* 821 | *Rvi6* | SSR | 1 | 0.0 | ^3^ |
| CH-Vf2 | *M. floribunda* 821 | *Rvi6* | SSR | 1 | 0.7 | ^3^ |
| M18-CAPS | *M. floribunda* 821 | *Rvi6* | CAPS | 1 | 0.2 | ^4^ |
| AL07-SCAR | *M. floribunda* 821 | *Rvi6* | SCAR | 1 | 0.9 | ^4^ |
| U01-SCAR | *M. floribunda* 821 | *Rvi6* | SCAR | 1 | / | ^5^ |
| AM19-SCAR | *M. floribunda* 821 | *Rvi6* | SCAR | 1 | 0.9 | ^6^ |
| D20 | *M. floribunda* 821 | *Rvi6* | SCAR | 1 | / | ^5^ |
| C09 | *M. floribunda* 821 | *Rvi6* | RAPD | 1 | 14 | ^5^ |
| ACS | *M. floribunda* 821 | *Rvi6* | AFLP | 1 | 0.2 | ^7^ |
| OPM18 | *M. floribunda* 821 | *Rvi6* | SCAR | 1 | 0.4 | ^7^ |
| OPAM19 | *M. floribunda* 821 | *Rvi6* | SCAR | 1 | 0.2 | ^7^ |
| OPAL07 | *M. floribunda* 821 | *Rvi6* | SCAR | 1 | 0.2 | ^7^ |
| M18 | *M. floribunda* 821 | *Rvi6* | SNP | 1 | 0.5 | ^8^ |
| ARD-CAPS | *M. floribunda* 821 | *Rvi6* | CAPS | 1 | 2 | ^2^ |
| T6-SCAR | *Malus baccata* jackii | *Rvi 11* | SCAR | 2 | 3.9 | ^9^ |
| CH05e03 | *M. baccata* jackii | *Rvi 11* | SSR | 2 | 0.6 | ^9^ |
| Z13 | *M. baccata* jackii | *Rvi 11* | SCAR | 2 | 3.3 | ^9^ |
| CH-Vbj3 | *M. baccata* jackii | *Rvi 11* | SSR | 2 | 1.5 | ^10^ |
| CH-Vbj1 | *M. baccata* jackii | *Rvi 11* | SSR | 2 | / | ^10^ |
| FBsnRvi11-1 | A722–7 | *Rvi11* | SNP | 2 | 2.8 | ^10^ |
| FBsnRvi11-2 | A722–7 | *Rvi11* | SNP | 2 | 0.8 | ^10^ |
| CH02f06 | GMAL 2473 | *Rvi4* | SSR | 2 | / | ^11^ |
| ARGH17 | GMAL 2473 | *Rvi4* | RGA | 2 | 0.3 | ^12^ |
| EA35MA41(262) | GMAL 2473 | *Rvi4* | AFLP | 2 | 0 | ^13^ |
| EA37MA39(188) | GMAL 2473 | *Rvi4* | AFLP | 2 | 0 | ^13^ |
| EA42MA39(500) | GMAL 2473 | *Rvi4* | AFLP | 2 | 6 | ^13^ |
| CH02f06 | GMAL 2473 | *Rvi4* | SSR | 2 | 6.9 | ^13^ |
| 9c10T7 | GMAL2473 | *Rvi4* | SNP | 2 | 0.7 | ^10^ |
| 21k14T7 | GMAL 2473 | *Rvi4* | SNP | 2 | 0.2 | ^10^ |
| FBsnRvi15-1 | GMAL 2473 | *Rvi4* | SNP | 2 | 0 | ^10^ |
| R_32981524_Lg2. | Russian apple TSR34T15 | *Rvi2* | SNP | 2 | 42.4 | ^14^ |
| OPL19 | Russian apple TSR34T15 | *Rvi2* | SCAR | 2 | 1.2 | ^15^ |
| CH02b10d | Russian apple TSR34T15 | *Rvi2* | SSR | 2 | 1.6 | ^16^ |
| FBsnRvi2-7 | Russian apple | *Rvi2* | SNP | 2 | 1.2 | ^10^ |
| FBsnRvi2-8 | Russian apple | *Rvi2* | SNP | 2 | 1.2 | ^10^ |
| CH02c02a | Russian apple TSR33T239 | *Rvi4* | SSR | 2 | 5.0 | ^11^ |
| FBsnRvi4-1 | TSR33T239 | *Rvi4* | SNP | 2 | 0 | ^10^ |
| ARGH37 | GMAL 2473 | *Rvi4* | SNP | 2 | < 1.5 | ^12^ |
| TNL1 | GMAL 2473 | *Rvi4* | SNP | 2 | 0.2 | ^12^ |
| OPL19e | B45 | *Rvi8* | SCAR | 2 | 1.3 | ^17^ |
| NZscAS07 | *Malus* × *robusta* | *Rvi16* | SCAR | 3 | / | ^18^ |
| NZscAN01 | *M.* × *robusta* | *Rvi16* | SCAR | 3 | / | ^18^ |
| NZmsCN943818 | *M.* × *robusta* | *Rvi16* | SCAR | 3 | 5 | ^18^ |
| Hi03d06 | *M.* × *robusta* | *Rvi16* | SSR | 3 | / | ^19^ |
| AU223667 | *M.* × *robusta* | *Rvi16* | SSR | 3 | / | ^19^ |
| Hi08e04 | ‘Geneva’ | *Rvi3* | SSR | 4 | / | ^20^ |
| CH02c02b | ‘Geneva’ | *Rvi3* | SSR | 4 | / | ^20^ |
| CH05d02 | ‘Fiesta’ | *Rvi3* | SSR | 4 | / | ^11^ |
| GDsnp14084 | ‘Geneva’ | *Rvi3* | SNP | 4 | 19.4 | ^21^ |
| GDsnp22370_F2 | ‘Geneva’ | *Rvi3* | SNP | 4 | 14.8 | ^21^ |
| TsuGNH076 | ‘Geneva’ | *Rvi3* | SSR | 4 | / | ^20^ |
| HB09-SSR | ‘Dülmener Rosenapfel’ | *Rvi14* | SSR | 6 | 4.0 | ^22^ |
| CH03d07 | ‘Dülmener Rosenapfel’ | *Rvi14* | SSR | 6 | 12.6 | ^22^ |
| NZ23g04 | ‘Dülmener Rosenapfel’ | *Rvi14* | SSR | 6 | 22.5 | ^22^ |
| CH01e12 | *M. floribunda* 821 | *Rvi7* | SSR | 8 | 10 | ^23^ |
| CH04f03 | ‘Durello di Forli’ | *Rvi13* | SSR | 10 | / | ^24^ |
| CH02b07 | ‘Durello di Forli’ | *Rvi13* | SSR | 10 | 9.0 | ^24^ |
| 9N6 | 1980-015-025 | *Rvi18* | Dart | 11 | 10.9 | ^25^ |
| Hi07d11 | 1980-015-025 | *Rvi18* | SSR | 11 | 5 | ^25^ |
| AFLP 362 | 1980-015-025 | *Rvi18* | AFLP | 11 | less than 0.9 | ^25^ |
| AFLP 314 | 1980-015-025 | *Rvi18* | AFLP | 11 | less than 0.9 | ^25^ |
| Rvi-SSR-476/478 | 1980-015-025 | *Rvi18* | SSR | 11 | / | ^25^ |
| Vg12-SSR | ‘Golden Delicious’ | *Rvi1* | SSR | 12 | < 0.25 | ^26^ |
| Vg15-SSR | ‘Golden Delicious’ | *Rvi1* | SSR | 12 | < 0.25 | ^26^ |
| Vg4-SSR | ‘Golden Delicious’ | *Rvi1* | SSR | 12 | < 0.25 | ^26^ |
| Vg14-SSR | ‘Golden Delicious’ | *Rvi1* | SSR | 12 | < 0.25 | ^26^ |
| MC105 | ‘Golden Delicious’ | *Rvi1* | RFLP | 12 | 3.0 | ^27^ |
| Hi07f01 | Hansen's baccata #2 | *Rvi12* | SSR | 12 | 13.7 | ^28^ |
| CH01f02 | Hansen's baccata #3 | *Rvi12* | SSR | 12 | / | ^28^ |
| Hi02d05 | Hansen’s Baccata #2 | *Rvi12* | SSR | 12 | 7.8 | ^28^ |
| Hi07h02 | *Malus micromalus* | *Rvi5* | SSR | 17 | 0.0 | ^29^ |
| OPB12(687) | *M. micromalus* | *Rvi5* | SCAR | 17 | 6 | ^30^ |
| OPB12 | *M. micromalus* | *Rvi5* | SCAR | 17 | 5.4 | ^30^ |
| OPB12 | *M. micromalus* | *Rvi5* | STS | 17 | 5 | ^30^ |
| FMACH_Vm2 | 9-AR2T196 | *Rvi5* | SSR | 17 | < 1 | ^31^ |
| FMACH_Vm3 | 9-AR2T196 | *Rvi5* | SSR | 17 | 0 | ^31^ |
| FMACH_Vm4 | 9-AR2T196 | *Rvi5* | SSR | 17 | < 1 | ^31^ |
| Vm-SCAR1 | 9-AR2T196 | *Rvi5* | SCAR | 17 | 0 | ^31^ |
| Vm-SNP5 | 9-AR2T196 | *Rvi5* | SNP | 17 | 0 | ^31^ |
| CH05d08 | 9-AR2T196 | *Rvi5* | SSR | 17 | 1 | ^31^ |
| Hi07h02 | 9-AR2T196 | *Rvi5* | SSR | 17 | 0 | ^31^ |

1.	Hemmat, M., Brown, S. K., Aldwinckle, H. S., Mehlenbacher, S. A. & Weeden, N. F. Identification and mapping of markers for resistance to apple scab from ‘Antonovka’ and ‘Hansen’s baccata #2’. *Acta Hortic.* **622**, 153–161 (2003).

2.	Dunemann, F. & Egerer, J. A major resistance gene from Russian apple ‘Antonovka’ conferring field immunity against apple scab is closely linked to the *Vf* locus. *Tree Genet. Genomes* **6**, 627–633 (2010).

3.	Vinatzer, B. A. *et al.* Isolation of two microsatellite markers from BAC clones of the *Vf* scab resistance region and molecular characterization of scab-resistant accessions in *Malus* germplasm. *Plant Breed.* **123**, 321–326 (2004).

4.	Tartarini, S., Sansavini, S., Vinatzer, B., Gennari, F. & Domizi, C. Efficiency of marker assisted selection (MAS) for the *Vf* scab resistance gene. *Acta Hortic.* **538**, 549–553 (2000).

5.	Gao, Z. S. & van de Weg, W. E. The *Vf* gene for scab resistance in apple is linked to sub-lethal genes. *Euphytica* **151**, 123–132 (2006).

6.	Tartarini, S., Gianfranceschi, L., Sansavini, S. & Gessler, C. Development of reliable PCR markers for the selection of the *Vf* gene conferring scab resistance in apple. *Plant Breed.* **118**, 183–186 (1999).

7.	Xu, M., Huaracha, E. & Korban, S. S. Development of sequence-characterized amplified regions (SCARs) from amplified fragment length polymorphism (AFLP) markers tightly linked to the *Vf* gene in apple. *Genome* **44**, 63–70 (2001).

8.	Patocchi, A., Gianfranceschi, L. & Gessler, C. Towards the map-based cloning of *Vf*: fine and physical mapping of the *Vf* Region. *Theor. Appl. Genet.* **99**, 1012–1017 (1999).

9.	Gygax, M. *et al.* Molecular markers linked to the apple scab resistance gene *Vbj* derived from *Malus baccata* jackii. *Theor. Appl. Genet.* **109**, 1702–9 (2004).

10.	Jänsch, M. *et al.* Identification of SNPs linked to eight apple disease resistance loci. *Mol. Breed.* **35**, 1–21 (2015).

11.	Liebhard, R. *et al.* Development and characterisation of 140 new microsatellites in apple (*Malus* x *domestica* Borkh.). *Mol. Breed.* **10**, 217–241 (2002).

12.	Galli, P., Patocchi, A., Broggini, G. A. L. & Gessler, C. The *Rvi15* (*Vr2*) apple scab resistance locus contains three TIR-NBS-LRR genes. *Mol. Plant-Microbe Interact.* **23**, 608–617 (2010).

13.	Patocchi, A., Bigler, B., Koller, B., Kellerhals, M. & Gessler, C. *Vr2*: A new apple scab resistance gene. *Theor. Appl. Genet.* **109**, 1087–1092 (2004).

14.	Baison, J. Mapping and identification of disease resistance candidate genes in three *Malus* populations using SSRs, DArT and Infinium SNP markers and Illumina sequencing technology. (University of the Western Cape, 2014).

15.	Bus, V. G. M. *et al.* The *Vh2* and *Vh4* scab resistance genes in two differential hosts derived from Russian apple R12740-7A map to the same linkage group of apple. *Mol. Breed.* **15**, 103–116 (2005).

16.	Baumgartner, I. O., Patocchi, A., Frey, J. E., Peil, A. & Kellerhals, M. Breeding elite lines of apple carrying pyramided homozygous resistance genes against apple scab and resistance against powdery mildew and fire blight. *Plant Mol. Biol. Report.* **33**, 1573–1583 (2015).

17.	Bus, V. G. M. *et al.* The *Vh8* locus of a new gene-for-gene interaction between *Venturia inaequalis* and the wild apple *Malus sieversii* is closely linked to the *Vh2* locus in *Malus pumila* R12740-7A. *New Phytol.* **166**, 1035–1049 (2005).

18.	Bus, V. G. M. *et al.* Genome mapping of an apple scab, a powdery mildew and a woolly apple aphid resistance gene from open-pollinated mildew immune selection. *Tree Genet. Genomes* **6**, 477–487 (2010).

19.	Peil, A. *et al.* Strong evidence for a fire blight resistance gene of *Malus robusta* located on linkage group 3. *Plant Breed.* **126**, 470–475 (2007).

20.	Perchepied, L. *et al.* Genetic mapping and pyramiding of two new pear scab resistance QTLs. *Mol. Breed.* **35**, (2015).

21.	Bastiaanse, H. *et al.* Scab resistance in ‘Geneva’ apple is conditioned by a resistance gene cluster with complex genetic control. *Mol. Plant Pathol.* **17**, 159–172 (2016).

22.	Soufflet-Freslon, V., Gianfranceschi, L., Patocchi, A. & Durel, C.-E. Inheritance studies of apple scab resistance and identification of *Rvi14*, a new major gene that acts together with other broad-spectrum QTL. *Genome* **51**, 657–67 (2008).

23.	Khajuria, Y. P., Kaul, S., Wani, A. A. & Dhar, M. K. Genetics of resistance in apple against *Venturia inaequalis* (Wint.) Cke. *Tree Genet. Genomes* **14**, (2018).

24.	Tartarini, S. *et al.* Characterisation and genetic mapping of a major scab resistance gene from the old Italian apple cultivar ‘Durello di Forli’. *Acta Hortic.* **663**, 129–134 (2004).

25.	Soriano, J. M. *et al.* Fine mapping of the gene *Rvi18* (*V25*) for broad-spectrum resistance to apple scab, and development of a linked SSR marker suitable for marker-assisted breeding. *Mol. Breed.* **34**, 2021–2032 (2014).

26.	Cova, V. *et al.* High-resolution genetic and physical map of the *Rvi1* (*Vg*) apple scab resistance locus. *Mol. Breed.* **35**, (2015).

27.	Marić, S., Lukić, M., Cerović, R., Mitrović, M. & Bošković, R. Application of molecular markers in apple breeding. *Genetika* **42**, 359–375 (2010).

28.	Erdin, N. *et al.* Mapping of the apple scab-resistance gene *Vb*. *Genome* **49**, 1238–45 (2006).

29.	Patocchi, A. *et al.* Identification by genome scanning approach (GSA) of a microsatellite tightly associated with the apple scab resistance gene *Vm*. *Genome* **48**, 630–636 (2005).

30.	Cheng, F. S. *et al.* Development of a DNA marker for *V*<SUB>*m*</SUB>, a gene conferring resistance to apple scab. *Genome* **41**, 208–214 (1998).

31.	Cova, V. *et al.* Fine mapping of the *Rvi5* (*Vm*) apple scab resistance locus in the `Murray’ apple genotype. *Mol. Breed.* **35**, 200 (2015).
